# Biosorption as a Perfect Technique for Purification of Wastewater Contaminated with Ammonia

**DOI:** 10.1007/s12010-021-03794-4

**Published:** 2022-05-28

**Authors:** Ibrahim Abdelfattah, Fathy A. El-Saied, Ali A. Almedolab, A. M. El-Shamy

**Affiliations:** 1 Water Pollution Research Department, National Research Centre, El-Bohouth St. 33, Dokki, P.O. 12622, Giza, Egypt; 2 Chemistry Department, Faculty of Science, Menoufia University, Shibin Al Kawm, Egypt; 3 General Hospital of Sadat City, Menoufia, Egypt; 4 Electrochemistry and Corrosion Lab, Physical Chemistry Department, National Research Centre, El-Bohouth St. 33, Dokki, P.O. 12622, Giza, Egypt

**Keywords:** Biosorption process, Waste management process, Batch adsorption study, Wastewater effluents, Modeling accuracy

## Abstract

*Eichhornia crassipes* root powder (ECRP) has been used to remove ammonia from aqueous solutions. The biosorption factors such as biosorbent dosage, pH, initial ammonia concentration, and contact time have been considered in batch conditions. The optimal conditions, at pH (6), sorbent dose 5 g/l, time (30 min) ammonia concentration (10 mg/l). Langmuir is better suited than Freundlich isotherm. The kinetic models Thomas, Yoon-Nelson, and Bohart-Adams were applied. These models showed that the adsorption capacity decreased with flow rate increases as follows: 32.57, 31.82, 31.25, and 30.17 mg/g, respectively, at a flow rate 10, 15, 20, and 25 ml/min. The root powder of *Eichhornia crassipes* was used to treat specific drainage wastewater obtained from the Sabal drain at Menoufia, Egypt. The average efficiency of ammonia removal was 87% per batch adsorption method at pH value = 7.5, sorbent dose 5 g/l, uptake period (30 min), and primary load 7.1 mg/l; however, ammonia removal by column continuous adsorption method exceeded 94%. In addition, ECRP is efficient in removing arsenic, sulfate, nitrates, nitrite, silica, iron, manganese, copper, zinc, aluminum, and lead from actual sewage wastewater, in addition to removing more than 75% COD.

## Introduction

Water contamination is amongst the most urgent concerns of the moment. Nitrogen compounds are a major freshwater contaminant. Nitrogen contaminants such as synthetic nitrogen, ammonia, nitrite and nitrates, soluble ammonia (NH_3_), and positively charged ammonium ions (NH_4_^+^) occur in wastewater. Harmony in the aqueous interface between two sources of ammonia however according to the reversible reaction:

1 $${NH}_{3}\leftrightarrow {NH}_{4}^{+}$$

When the solution’s pH is less than 9.3, ammonia that is bound to hydrogen ions will yield ammonium ions to be dominant [1]. Total ammonia nitrogen (TAN) in an aqueous solution is equivalent to NH_3_ and NH_4_^+^ summation. The ingestion of high concentration of ammonia causes severe and chronic effects on human health including eyes, nose, and mouth; skin inflammation; and burns and reduces insulin sensitivity, causing the blue baby syndrome, permanent blindness, and lung or death [2–4]. High concentrations of NH_3_ and NH_4_^+^ in water supplies increased the need for oxygen and disrupted marine life, which is harmful to fish with very small concentrations of around 0.2 mg/l. Ammonia is toxic to all vertebrates that induce epilepsy, coma, and cell death in the central nervous system; cell death in the central nervous system is caused by redistribution of potassium (K^+^) with elevated NH_4_^+^ concentration that threatens to depolarize neurons [4–6]. Many technologies have been used to remove ammonia from wastewater, such as chemical, biological, and adsorption. Nitrification (by aerobic bacteria)/denitrification (by anaerobic bacteria) is a biological mechanism for extracting ammonia from urban and industrial wastewater, but at higher concentrations of ammonia, the cycle is impaired due to the toxic effect of ammonia on nitrifying bacteria [7]; Fawzy et al., 2018; Abdelfattah 2018; Ibrahim et al., 2016a; [8], El-Shafai et al., 2016; El-Awady et al., 2015; [6, 9]. Uranbileg Daalkhaijav [10] announced 70 % elimination of nitrification/denitrification ammonia from wastewater (Uranbileg and [5, 10]. Reza et al. [11] recorded nitrification/denitrification removal efficiency of ammonia at various concentrations 25, 40, 80, 120, and 160 mg/l which are 87 %, 89 %, 72 %, 66 %, and 62 % [11, 12]. Some experiments have used algae to remove elevated ammonia concentrations from wastewater. The *Scenedesmus* sp. black algae have been able to absorb ammonia effectively in concentrations up to 100 mg/l. Halfhide et al. used ammonia reduction microalgae, with 65 % elimination [13–16]. The ozone molecule’s direct oxidation of ammonia is relatively sluggish and produces nitrate, which hence does not remove absolute nitrogen. Xianping Luo et al. [17] announced the ozonation elimination of 85 % of ammonia (Xianping [17],Uranbileg and [10]. Zong et al. (2017 recorded 28.5 % ozonation extraction of total nitrogen [18], Reza et al, 2010. An ion-exchange mechanism is the fusion of liquid phase ions of equal charge with electrostatically bound ions to an insoluble layer of resin.

2 $$B+A\leftrightarrow B+{A}^{-}$$

Malovanyy et al. [19] recorded 88% elimination of zeolite-based ammonium ions [19, 20]. Malekian et al. [21] reported the elimination of ammonium ions by natural Iranian zeolite by 91.5 % [16, 21]. Saltalý et al [22] recorded 75–83 % ammonium ion removal using natural Turkish zeolite [17, 22]. Adsorption is an efficient and cost-effective system for eliminating ammonium ions and versatile in nature; in many cases, high-quality treated effluent and adsorbents may be regenerated by an acceptable desorption cycle. Biosorption has been used in recent years as a natural adsorbent with higher performance, low cost, quality, and fast application in the removal of ammonia from the atmosphere [18, 19, 21–23]. Biosorption of ammonium ions from aqueous solutions is a very promising method for eliminating pollutants from the ammonium ions. Table 1 is a compilation of literature on the elimination of ammonium ions by the adsorption process. In this study, *Eichhornia crassipes* root powder (ECRP) from *Eichhornia crassipes* (water hyacinth) was used to remove ammonia from synthetic and real wastewater collected from Sabal drain at Menoufia, Egypt, using adsorption methods for batch and column. A concentration of ammonia ranged between 5.4 and 8.2 mg/l in the Sabal drain. The main source of this pollutant is the municipal wastewater discharged through septic tanks in the surrounding villages, although the permissible discharging limits to the Nile water are less than 0.5 mg/l.

**Table 1:** The summary of reports on ammonium ion removal by adsorption method

Biosorbent	Maximum adsorption capacity (mg/g)	Removal %	References
*Posidonia oceanica* (L.) fibers	1.8	-	23
Activated sludge	88	95	24
*Microbacterium* sp.	-	91.3	25
Supported Pt catalysts	-	97.5	26
Ozone	-	99	28
Macro-algae	0.3	70	29
GAC-sand dual media filter	-	45	30
Novel acryl biofilm carrier material	-	98.5	31
Ammonia volatilization	-	99	32
Activated carbon	1.8	-	33
Modified chitosan	-	82.1	34
Zeolite synthesized from fly ash	24	-	35
Sawdust	1.7	-	36

### Sources of Ammonia

Agriculture is the major source of ammonia pollution, as it is emitted through manure and slurry, as well as the usage of synthetic fertilizers. Agricultural sources accounted for 82% of all ammonia emissions in the UK in 2016. Trace quantities of ammonia are also emitted from several sources, including landfills, sewage treatment facilities, car emissions, and industry [24] Surface water eutrophication is produced mostly by nitrogen and phosphorus pollution from industrial effluent, agricultural fertilizer, and urban sewage, which has long been a source of worry in many countries. These nutrients cause a range of problems, such as toxic algal blooms, oxygen depletion, fish fatalities, biodiversity loss, loss of aquatic plant beds and coral reefs, and other problems [25]. The landfills are considered a low-cost technique, but there are a few bit disadvantages such as the formation of leachate including organic chemicals, NH_4_^+^, and N-containing compounds [26]

## Materials and Method

### Materials

**Fig. 1:**
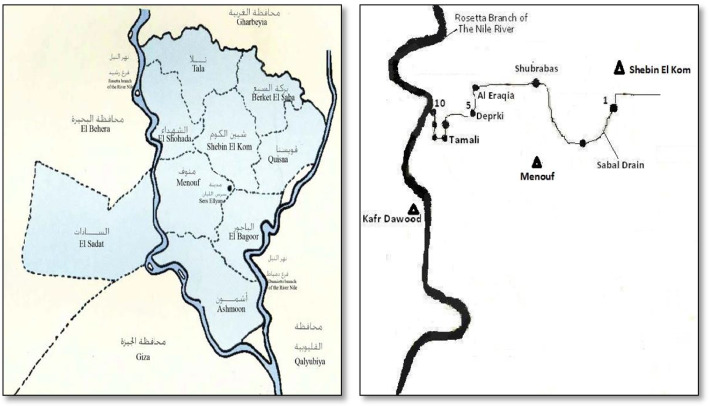
Location and sources of ammonia pollutants mainly come from agricultural activities and also illegal municipal wastewater draining

Ammonia stack solutions were prepared by dilution of ammonia solution 25% prepared by Sigma-Aldrich. pH ideals of prepared contaminated water were accustomed for the chosen number using 1 M of hydrochloric acid and 1 M of sodium hydroxide. Real drainage wastewater samples were collected from Sabal drain at Menoufia, Egypt, and the location is expressed in Fig. 1.

### Equipment

The suspension solutions were shaken by lab shaker, WiseShake, SHO-2D, South Korea. WTW-inolab, Germany, ECRP. The obtained imageries were carried out by SEM. The pictures at diverse amplifications applying Quanta-250 FEG, USA. The absorption spectra of FTIR or the ECLP have documented between 400 and 4000 ranges applying Jasco FTIR spectroscopy, Japan. The concentrations of ammonia were measured using American Standard Methods [27].

### Preparation of the Biosorbent

**Fig. 2:**
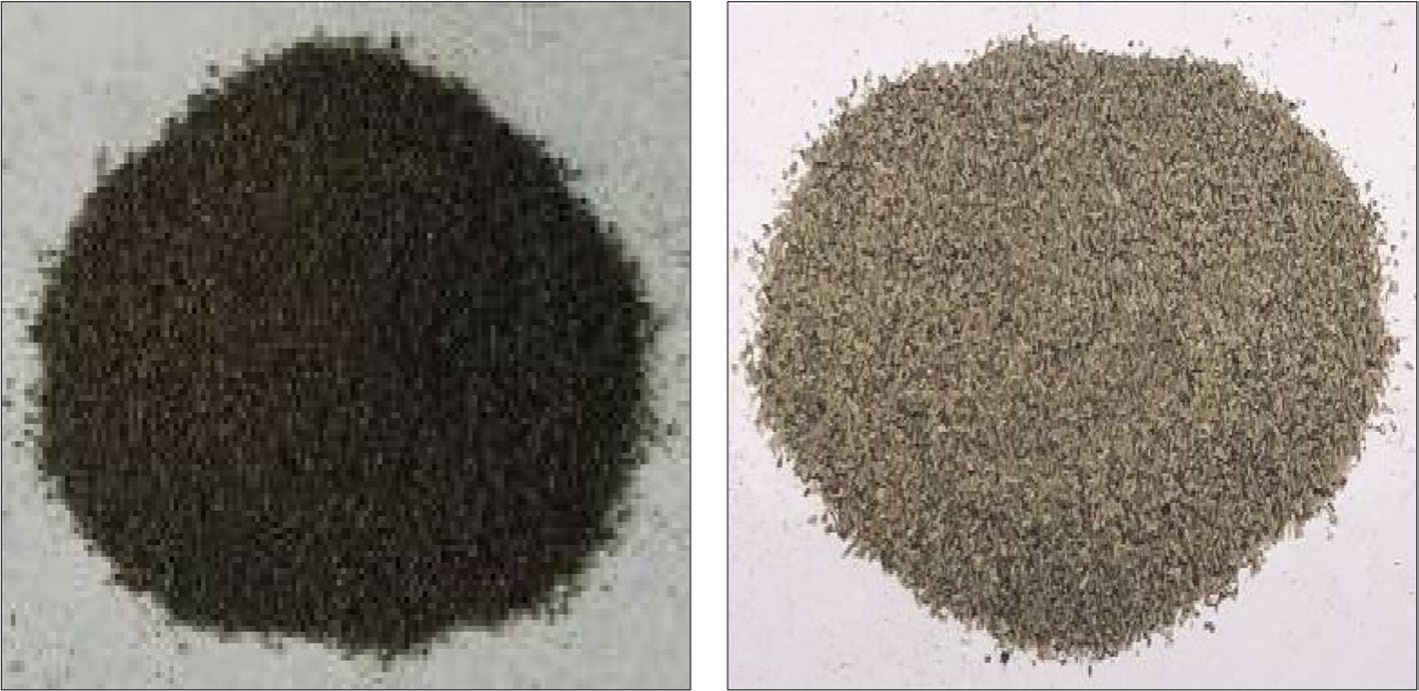
The biosorbent materials in the final form before application of batch and continuous flow trials. The left one is colored sample, and the right one is the decolored sample

*Eichhornia crassipes* (water hyacinth) was collected from the Rosetta branch of the Nile River at the governorate of Menoufia, Egypt. The clean biosorbent is dried in an oven at 80 °C for 12 h. The dried form is crushed in a laboratory mill and then sieved to a similar particle size. The biosorbent is washed again with deionized water, and the decolorization process is subjected by washing with HCl and then NaOH (see Fig. 2). The decolored form is washed again by deionization water and then dried at 80 °C for 1 day.

### Methodology

Intended for batch method, freshly prepared solutions of ammonia with known initial concentration were used for biosorption experiments. Various biosorbent doses were immersed in 100 ml of the synthetic contaminant solution. The experiments were stirred employing a lab shaker with 250 rpm for 5–60 min. Whatman qualitative No. 4 filter paper was used to separate biosorbents from the solutions. The parameters of untreated and treated wastewaters have been investigated corresponding toward techniques of the American Standard Methods [27]. The range of ammonia in the drain in the area under study can be illustrated in Table 2. It represents the concentration of ammonia in different sites of the Sabal drain.

**Table 2:** NH_3_ concentration in water at different sites of Sabal drain

Site no	NH_3_ value mg/l			Min	Max	Average
	Sample I	Sample II	Sample III			
1	5.6	4.2	5.5	4.2	5.6	5.1
2	8.4	4.2	5.3	4.2	8.4	6.0
3	2.8	2.5	2.4	2.4	2.8	2.6
4	25.7	20.7	26.5	20.7	25.7	24.3
5	23.5	26.0	22.3	22.3	26	23.9
6	2.0	9.3	8.5	2	9.3	6.6
7	19.0	18.0	15.6	15.6	19	17.5
8	28.0	24.6	26.5	24	28	26.4
9	9.1	10.5	9.8	9.1	10.5	9.8
10	7.3	6.7	7.5	6.7	7.5	7.2

#### Batch and Continuous Column Experiments

**Fig. 3:**
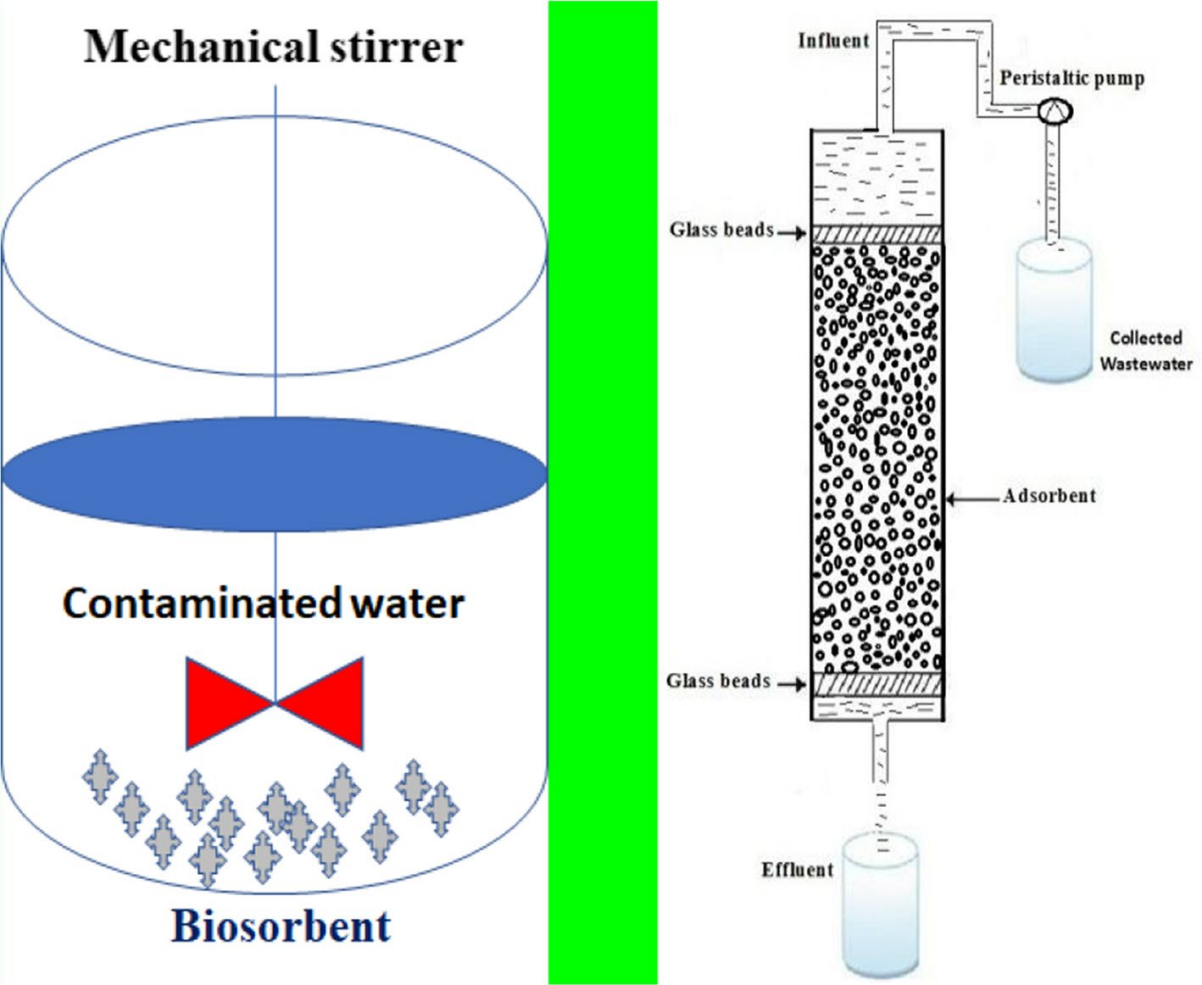
Batch and continuous column experiments

Figure 3 shows the structure of the adsorption column, and the research uses a bursting bed column made of polypropylene with an inner diameter of 5 cm, a height of 100 cm, and overall volume of 1.96 l. The column has glass beads with a diameter of 1.5 mm which were positioned at the top to achieve a height of 2 cm, and a 0.5 mm stainless sieve supported by glass beads was given at the bottom to support the packaging. A known quantity (200 g) of particle-sized *Eichhornia crassipes* (water hyacinth) powder (2.4–55.7 μm) was placed in the column to yield sorbent bed height (80 cm) and volume (1.57 l).

A peristaltic pump had fed upward ammonia solutions of initial concentration 10 mg/l at pH 7.3 to obtain desirable flow rates inside the column. Ammonia concentrations of the sewage at the column exit collected at different time intervals were analyzed, and the column system was operated till the effluent ammonia concentration reached equilibrium. From the results, at the beginning of contact between ammonia solution and biosorbent on the column, ammonia removal is high, then gradually declines, and then rises until it reaches equilibrium [28].

## Results and Discussions

### Biosorbent Investigation

#### Scanning Electron Microscope for Biosorbent

**Fig. 4:**
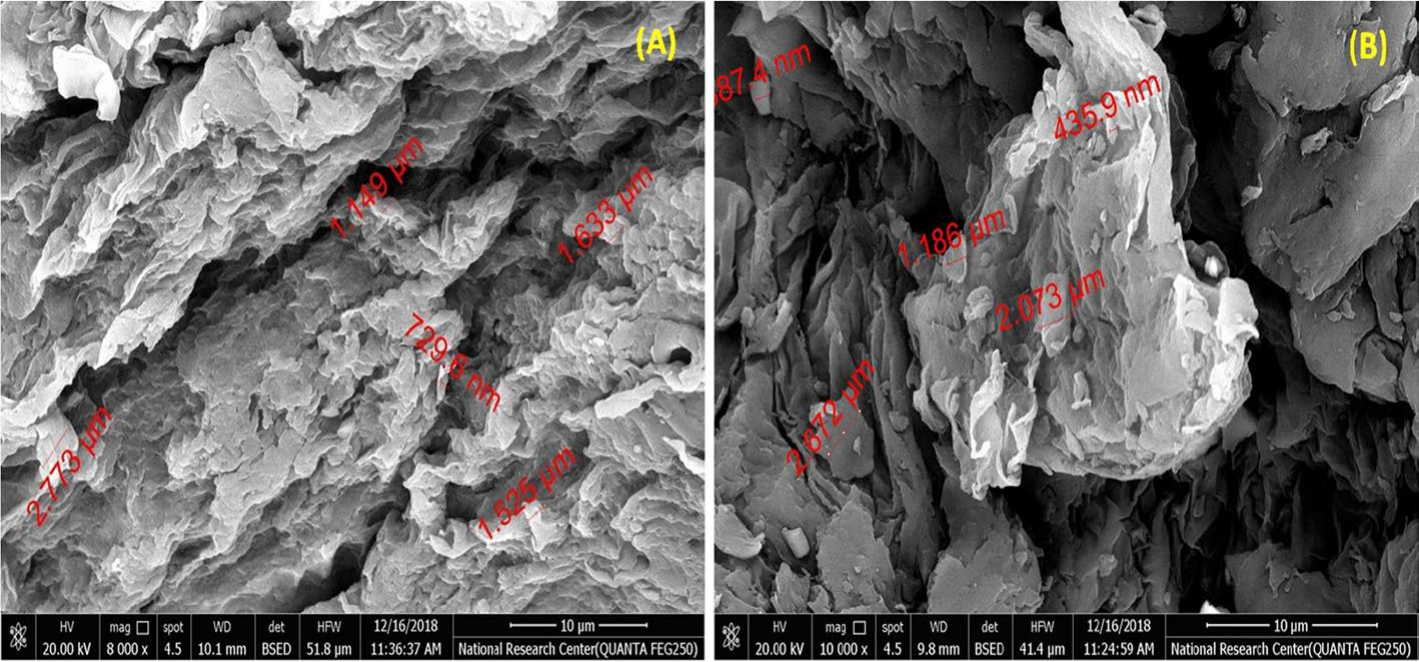
**A** The particle size of ECRP and **B** the pore size at the surface of ECRP

The superficial expanse of the biosorbent was evaluated by using a scanning electron microscope. Figure 4A shows the particle size of ECRP about 729.6 nm–2.77 μm, and Fig. 4B shows the pore size at the exterior of ECRP particles about 435.9 nm–2.67 μm. Figure 4A and B correspondingly reveal the used particle size and the dispersal of spongy composition alongside the outward of their ECRP before treatment.

#### FTIR Spectra of Biosorbent

**Fig. 5:**
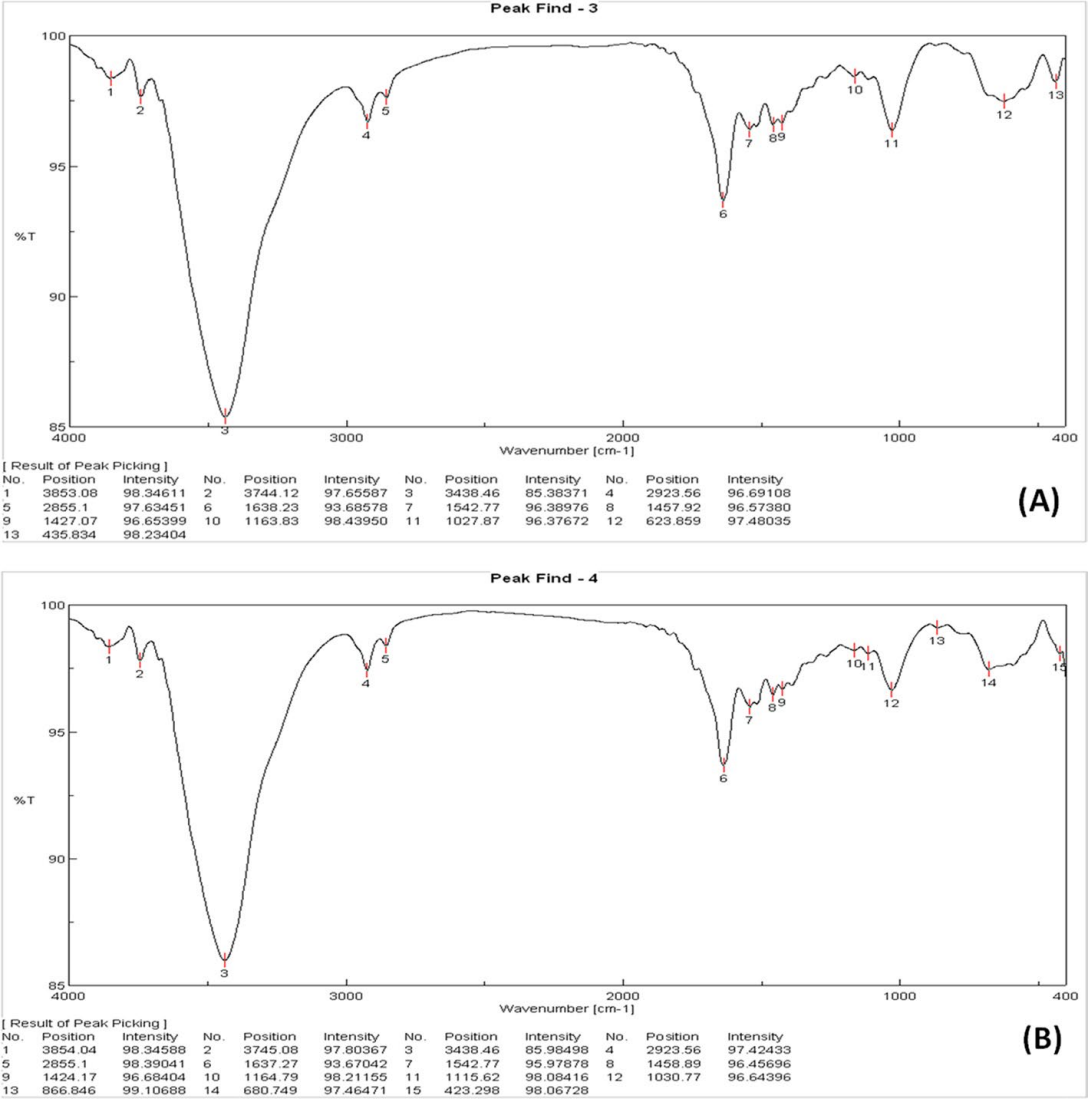
**A** FTIR spectrum of ECRP before treatment and **B** FTIR spectrum of ECRP after treatment

Figure 5A and B display the Fourier transform infrared (FTIR) spectrum of ECRP before and after treatment. The FTIR was applied to attain data about the possible adsorbent-ammonia interactions. The FTIR bands of the unloaded and the ammonia-loaded biosorbent are in the assortment range of 400- 4000 cm-1. The broad and strong peaks at 3853.08, 3744, and 3438 cm^−1^ represent OH stretching vibrations, while the peak observed at 2923 and 2855 cm^−1^ showed the asymmetric C-H aliphatic group. The strong peak at 1638 cm^−1^ was appointed to C = C extending, while the obtained peak at 1542 represents N–H bending, and the gained peak at 1457 and 1427 could be present in the C-H bending.

The gotten peak at 1027 cm−1 and 1163 assigned C-O stretching vibrations and the observed peaks at 623 and 435 showed C-X stretching (X = Cl, Br, F, or I). It was notified that most of these functional groups have capabilities to adsorb ammonia very well from the spectra, the strength of ammonia-loaded ECRP was slightly different than the spectra of ECRP before adsorption, and there were some shifts in wave numbers after adsorption.

### Adsorption Experiments

In this section, some parameters which affect the process of adsorption will be studied as follows:

#### Effect of Sorbent Dose

**Fig. 6:**
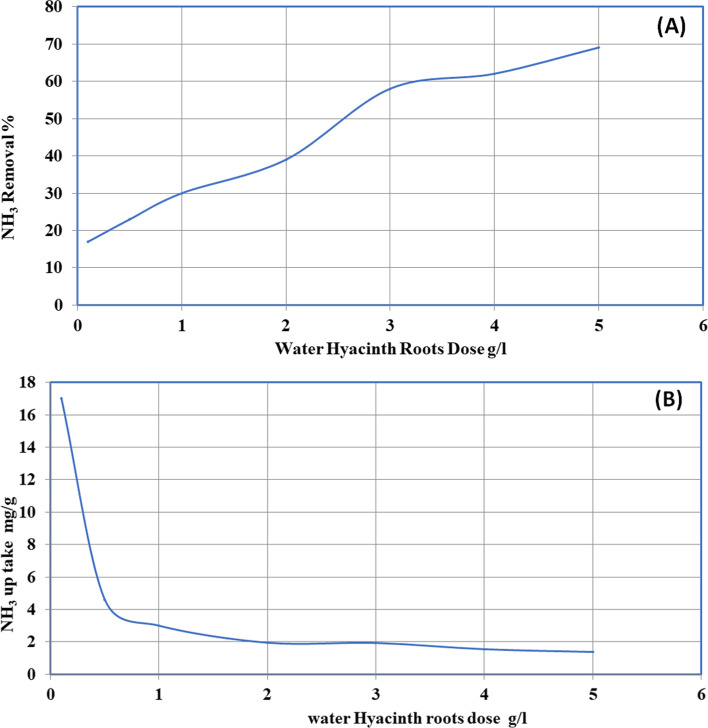
**A** Effect of ECRP dose on the removal of ammonia and **B** effect of ECRP dose on ammonia uptake

The consequence of ECLP amount on the elimination of NH_3_ was carried at varying doses (0.5, 1, 2, 3, 4, and 5 g/l) at pH 7.4, initial concentration of 10 mg/l, shaking speed of 250 rpm, and connection time of 60 min. Figure 6A shows that ammonia exclusion % improved with the growth of ECRP dosage. Figure 6B demonstrates that the ammonia exclusion diminished through growing ECRP dose that ascribed to the saturation of the active sites. Biosorption capacity declined with snowballing biosorbent dose for two reasons. First, with increasing biosorbent dose, aggregation of biosorbent particles leads to a decline in an entire superficial expanse of the biosorbent and an upsurge in dispersion path length. Furthermore, the growth in the dose of biosorbent at a steady concentration of ammonia and solution quantity will have an advantage to unsaturated active sites throughout the uptake procedure [29].

#### Consequence of Interaction Period

**Fig. 7:**
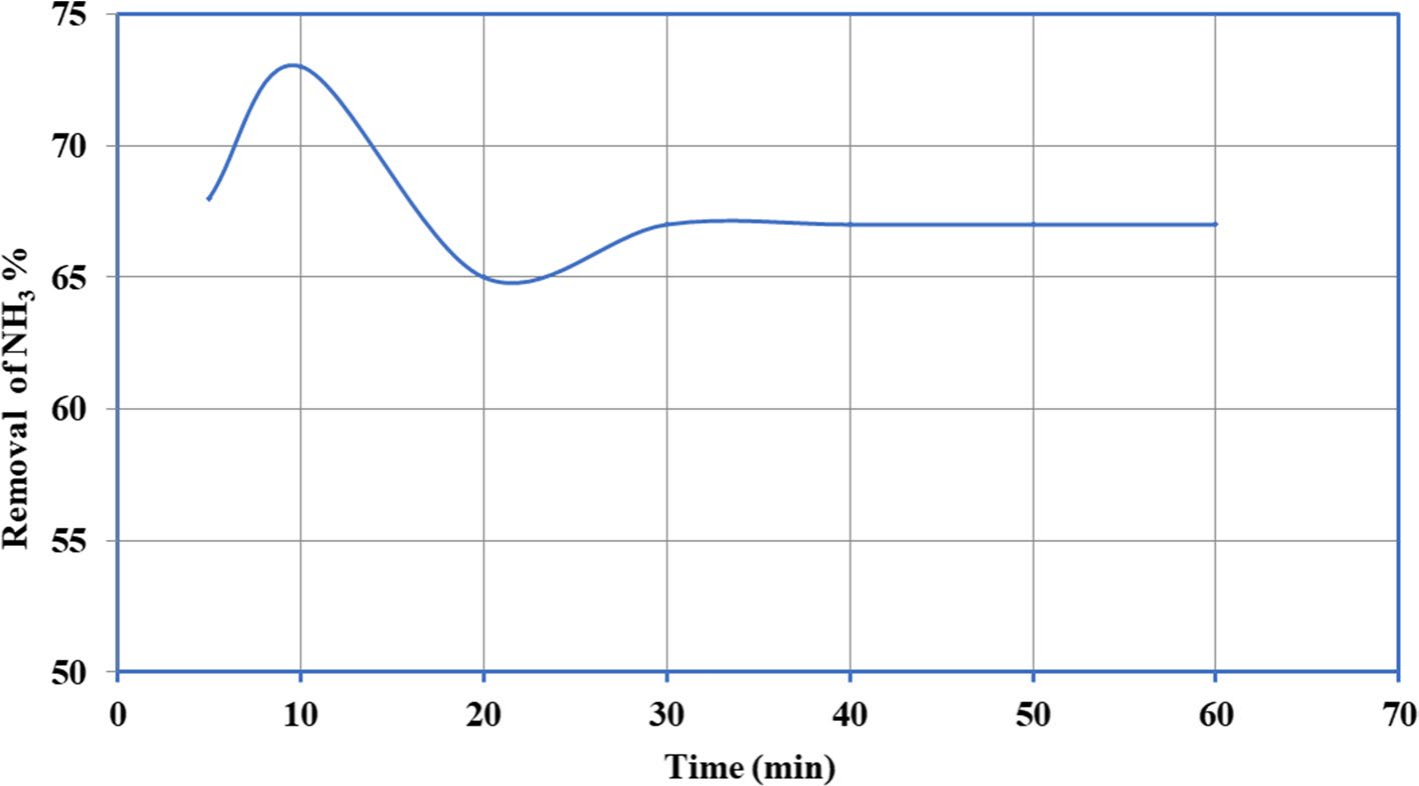
Effect of contact time on the removal of ammonia

The outcome of the connection period was studied at 5, 10, 20, 30, 40, 50, and 60 min on the removal percentage of NH_3_ by ECRP at pH (7.2), the dose of biosorbent (5 g/l), flow (250 rpm), and original concentration of ammonia (10 mg/l); the outcomes are publicized in Fig. 7. The proportion of elimination of NH_3_ was speedy in the initial 10 min but then develops gradually till achieving balance. The removal percentage at equipoise was 67% within 30 min. High ammonia removal was adsorbed in the first 10 min probably due to film diffusion on the external surface of the biosorbent when all adsorbent sites were vacant, and the gradient of the solute concentration was high [30].

#### Effect of pH

**Fig. 8:**
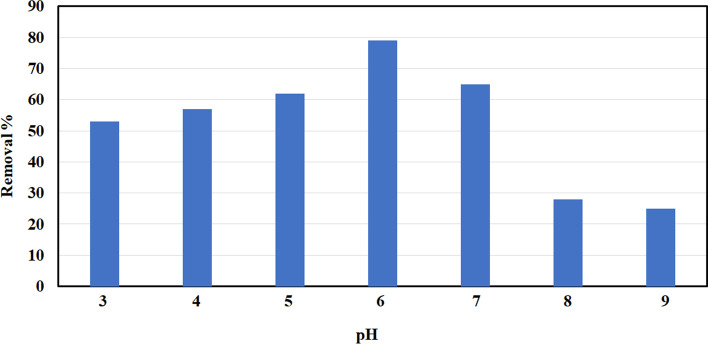
Effect of pH on ammonia removal

Effect of pH was studied at 3, 4, 5, 6, 7, 8, and 9 on the elimination of NH_3_ at a specific dose (5 g/l), preliminary ammonia concentration (10 mg/l), shaking speed (250 rpm), and interaction period (60 min). In addition, the solution pH has a significant impact on the uptake of NH_3_. Figure 8 displays that the extreme removal of ammonia was at pH 6. The previous studies stated that; the optimal pH for ammonia removal was at pH (5–6); the properties of ammoniacal solution explain the result; the existence of two types, NH_3_ (basic) and ammonium ions, NH_4_^+^ (acidic) [31–33]. Ammonia removal at low pH is high due to the cation exchange mechanism in an aqueous solution. However, ammonia removal decreases at pH < 5 because of H^+^ competition.

#### Effect of Original Concentration of Ammonia (C_0_)

**Fig. 9:**
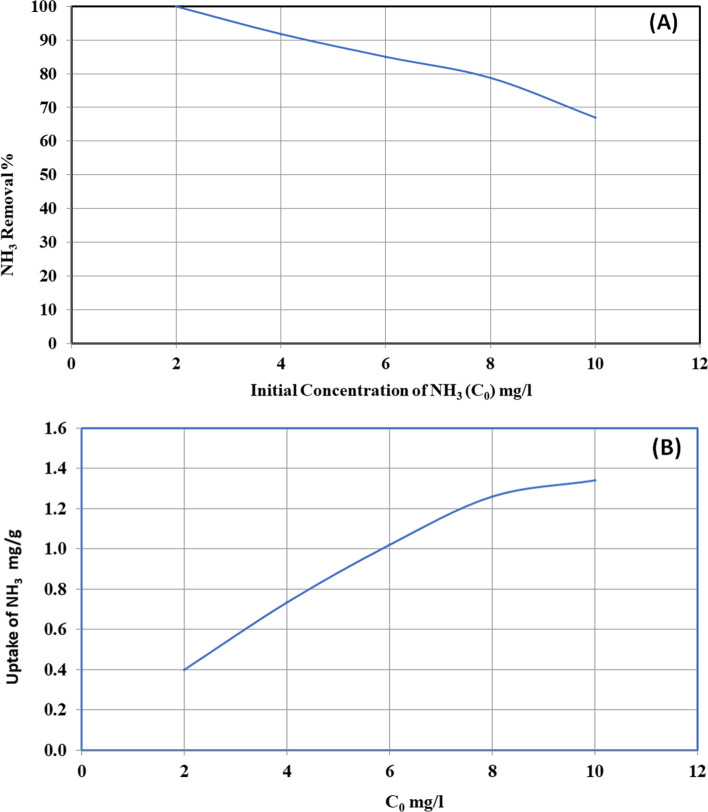
**A** Effect of initial concentration on the removal of ammonia and **B** effect of initial concentration on ammonia uptake

The behavior of ammonia uptake by ECRP was supported by using different initial ammonia concentrations (3, 4, 6, 8, and 10 mg/l) at optimum pH (6), interaction period (30 min), dosage (5 g/l), and flow (250 rpm). Figure 9A shows that the removal % of ammonia declines with the increase in its primary load, while along with growing loads of ammonia, the compulsory spots turn out to be extra rapidly drenched as the expanse of biosorbent concentration remained constant [34]. Figure 9B shows that ammonia uptake increases with the increase in its initial concentration.

#### Adsorption Isotherm

The adsorption isothermal equation defined the relationship between the aqueous phase concentration of the solute and the sum of the adsorbed solvent. The isotherms of adsorption are measured to identify the adsorption process [34]. The Freundlich and Langmuir isothermal equations of adsorption have been used effectively in numerous processes of adsorption [35–40].

#### Model of Langmuir Isotherm

This formula is predicated on the presumption that the maximal uptake correlates to a dense monolayer species of adsorbate on the surface of the adsorbent. The adsorption strength is unchanged due to the technological breakthroughs of adsorbents into the surface plane [7].

This formula is defined as:

3 $${q}_{e}=\frac{{q}_{\mathrm{max}} *{K}_{l} {C}_{e}}{1+{K}_{l} {C}_{e}}$$

The linearized form is:

4 $$\frac{{C}_{e}}{{q}_{e}}=\frac{1}{{q}_{m} {K}_{l}}+\frac{1}{{q}_{m}}*{C}_{e}$$

From equation (5)

5 $$Slope=\frac{1}{{q}_{m}}$$

6 $$Intercept=\frac{1}{{q}_{m} {K}_{l}}$$

where *q*_*m*_ and *K*_*L*_ are constants related to Langmuir respectively to the capability of adsorption and its energetic yield, *C*_*e*_ (mg/l) is concerned with the balance load, and *q*_*e*_ (mg/g) is the adsorption measurements at equilibrium.

The Langmuir dimensional showed less separation constant factor or balanced factors, *R*_*L*_, which is specified by the subsequent formula:

7 $${R}_{L}=\frac{1}{1+{K}_{L}* {C}_{0}}$$

**Fig. 10:**
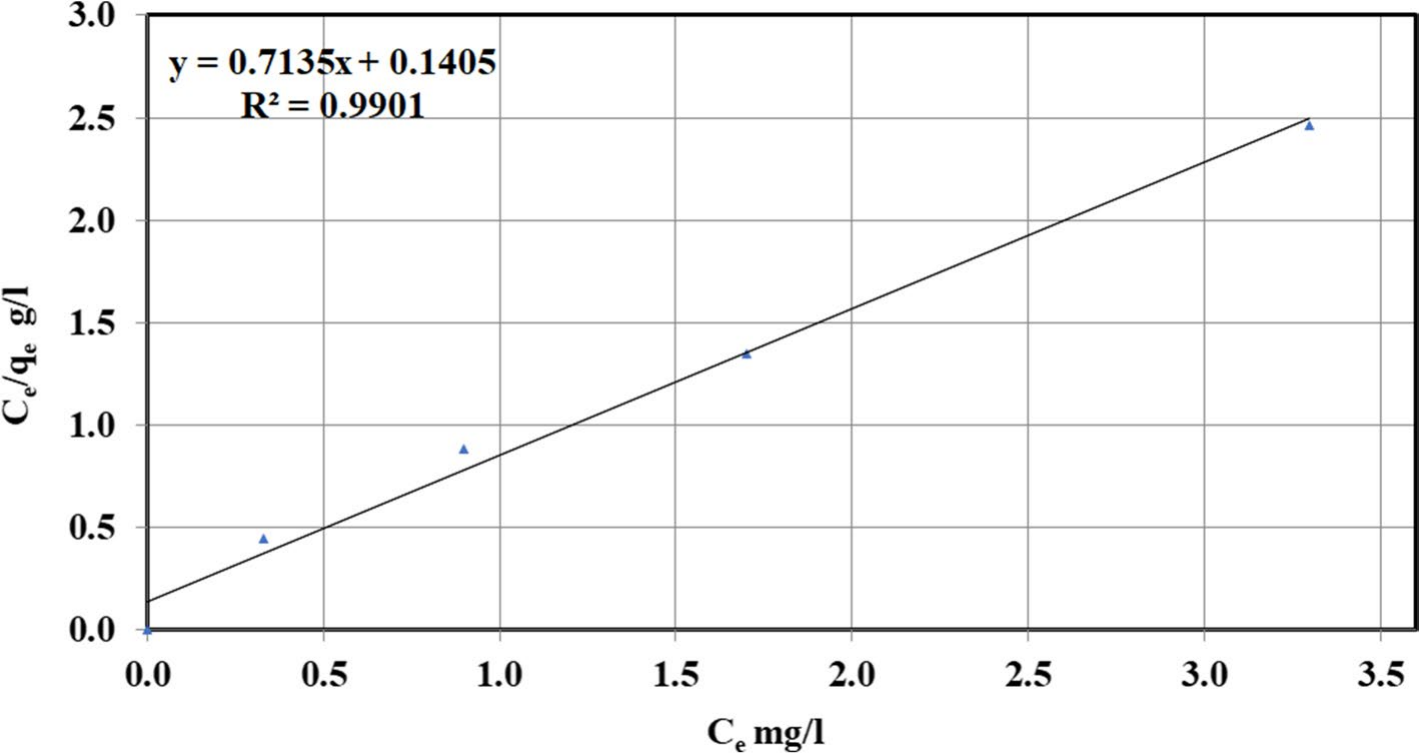
Langmuir plot of ECRP as adsorbent for NH3 removal

From the rate of RL, it can be considered and evaluated via the above expression, the physical meaning of the adsorption process to be any disadvantageous when (*R*_*L*_ > 1), straight at what time (*R*_*L*_ = 1), satisfactory while (0 < *R*_*L*_ < 1) and irreparable what (*R*_*L*_ = 0). The *R*_*L*_ assessments for the procedure of adsorption of NH_3_ with ECRP have magnitudes amongst 0 and 1, implying that the procedure of adsorption is favorable and a high value of *K*_*L*_ was given away to be a function of strong bonding between ammonia and biomass [8, 41–45]. The plotting of *C*_*e*_/*q*_*e*_ alongside *C*_*e*_ is revealed in Fig. 10. Removal of NH_3_ on ECRP yielded a straight-talking line. Constants of Langmuir isotherm and their correlation coefficients *R*^2^ are exposed in Table 3.

**Table 3:** Langmuir constants for the sorption of NH_3_ onto ECRP

Slope	Intercept	*q*_*m*_	*K*_*L*_	*R*_*L*_	*R*^2^
0.713	0.140	1.40	5.09	0.019–0.089	0.990

#### Freundlich Isotherm Model

Amongst the most popular technical explanations for isothermal adsorption is the Freundlich isotherm that offers an articulation concerning the conglomeration of the surface and the exponential dissemination of effective positions and their forces. The isotherm at Freundlich is described as:

8 $${q}_{e}={{C}_{e}}^\frac{1}{n}$$

**Fig. 11:**
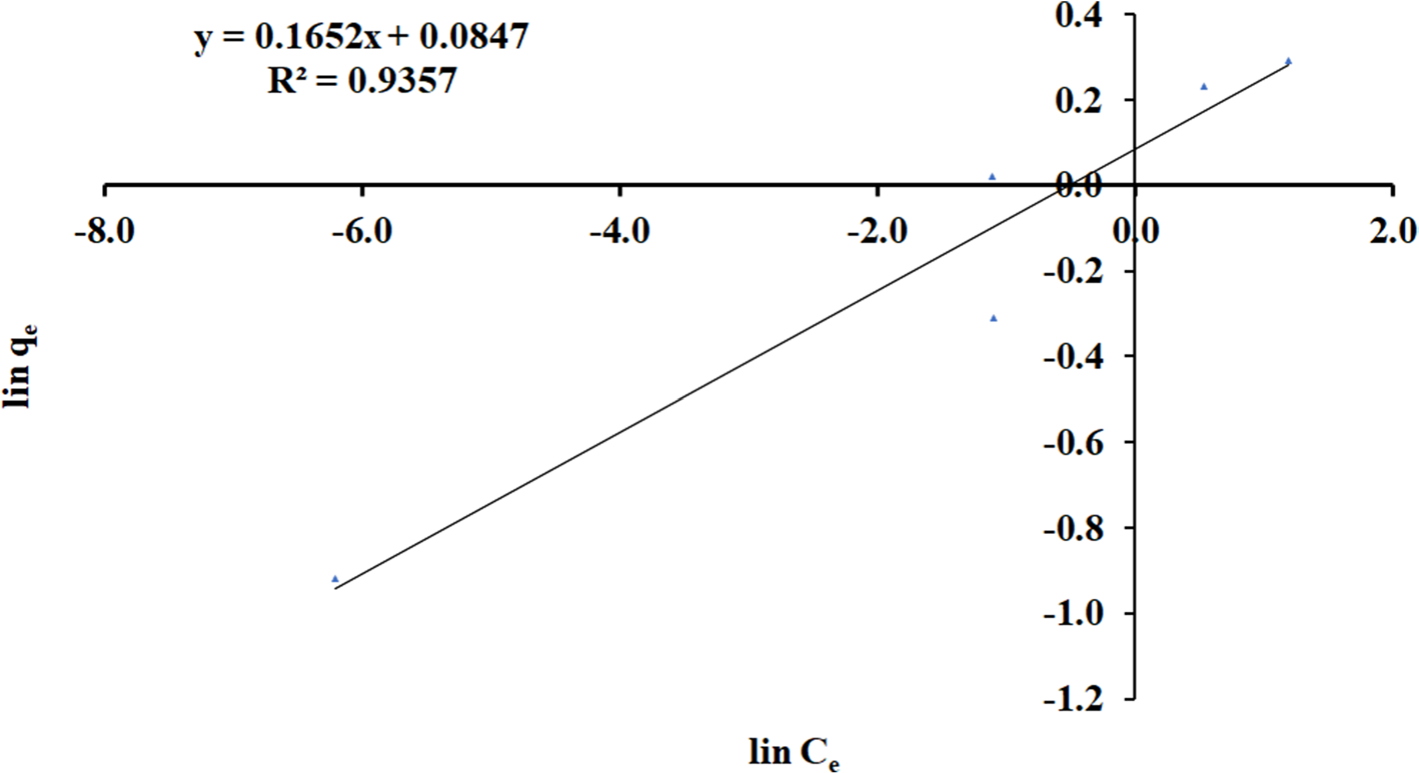
Plot of Freundlich isotherm for adsorption of NH3 on ECRP

And in linearized form is:

9 $$\mathit{ln}{q}_{e}=\mathit{ln}{K}_{F}+\left(\frac{1}{n}\right)\mathit{ln}{C}_{e}$$

10 $$Slope=\frac{1}{n}$$

11 $$Intercept=lin {K}_{F}$$

where *q*_*e*_ (mg/g) is the adsorption capacity at equilibrium, *C*_*e*_ (mg/l) is the ammonia load at equilibrium, *K*_*F*_ is a temperature-related constant, and *n* is the adsorption constant for the approach. The plotting of *ln q*_*e*_ versus *ln C*_*e*_ is given away in Fig. 11. The uptake of NH_3_ onto ECRP a straight line is provided which extinguishable for the standards of Freundlich constant (n) amongst 2 and 10 showed a decent removal capacity [8, 46]. The constants of Freundlich isotherm and their correlation coefficients *R*^2^ are exposed in Table 4.

**Table 4:** Freundlich constants for the sorption of ammonia onto ECRP

Slope	Intercept	*n*	Lin K_F_	*K*_*F*_	*R*^2^
0.165	0.084	6.061	0.084	1.088	0.935

### Flow Rate Consequence

**Fig. 12:**
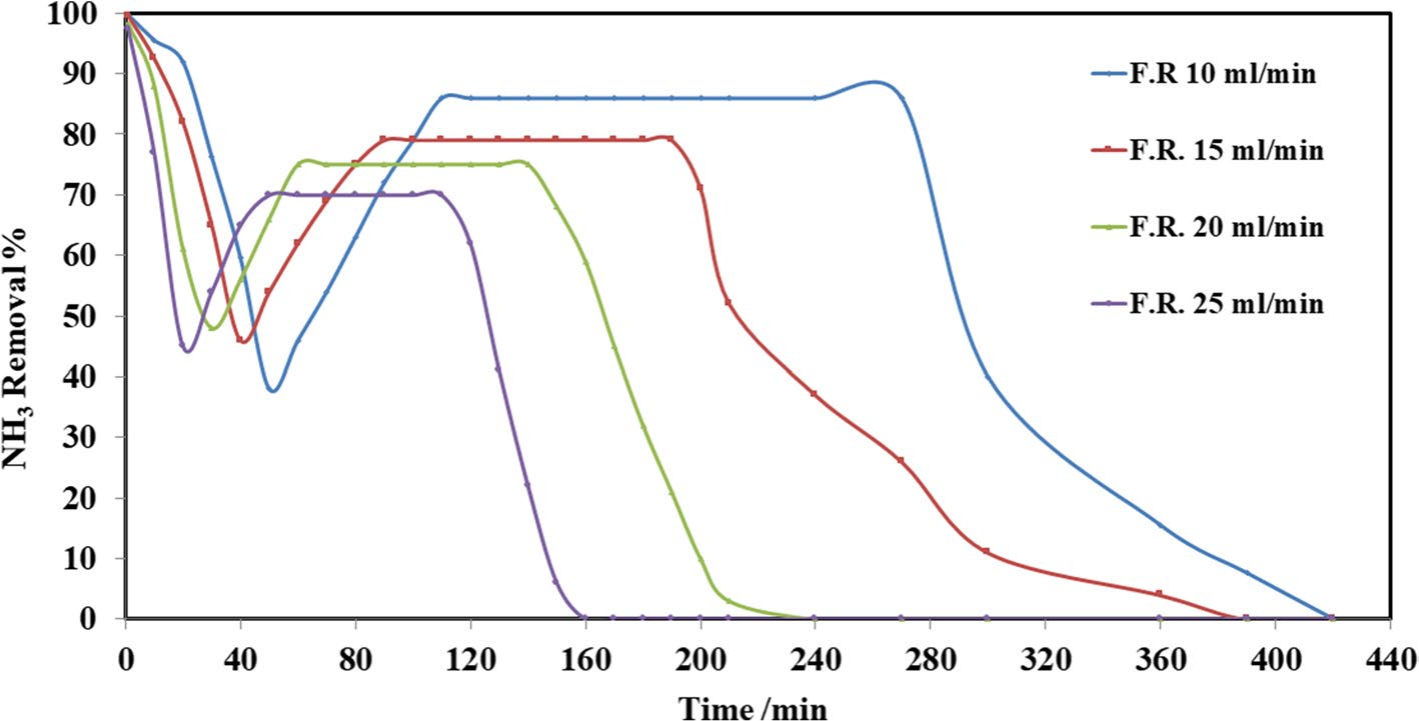
Effect of flow rate on ammonia removal by adsorption column

The study of flow rate effect on adsorption becomes an important factor [47]. In this work, the sorption capacity of *Eichhornia crassipes* powder is studied for various flow rates in the assortment of 10, 15, 20, and 25 ml/min for the original concentration of ammonia 10 mg/l and divan elevation of 80 cm. Figure 12 represents ammonia removal % against time for the rates of flow 5, 10, and 20 ml/min. Table 5 shows that ammonia removal % decreased by increasing the flow rate. The removal efficiency at steady state was 86%, 79%, 75%, and 70% for the rates of flow 10, 15, 20, and 25 ml/min, respectively. Figure 12 shows an increase in decreasing the flow rate through ammonia removal. The reduction in ammonia removal at higher flow rates is outstanding to the decrease of retention time for the solute to interact with the biosorbent and the restricted diffusion of particles into the adsorptive spots or holes of the biomass [48]. From the results, it was found that the rate of flow of 10 ml/min is the best in the elimination process. The important feature in the design of fixed-bed adsorption column is the rate of flow curve for the effluent, and mathematical models to fit them were applied in this study for the evaluation of column efficiency for the adsorption process [49–51].

**Table 5:** Thomas model parameters

Flow rate	*K*_Th_	*Q*_max_	*R*^2^
10	0.0035	32.7	0.987
15	0.0026	32.1	0.965
20	0.0032	31.3	0.946
25	0.0029	29. 7	0.921

### Adsorption Models in Continuous

#### Thomas’s Model

**Fig. 13:**
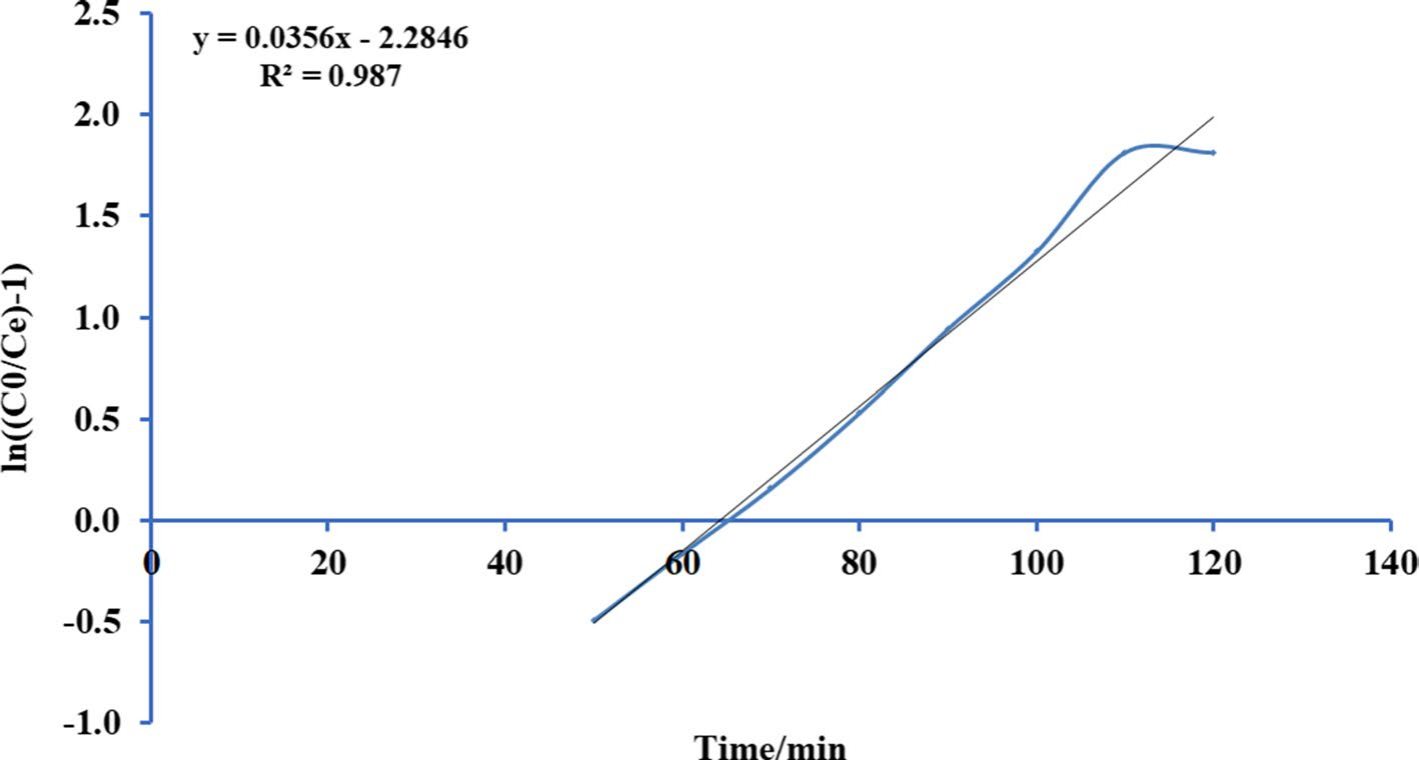
Plot Thomas mathematical model for ammonia adsorption by bed column at flow rate 10 ml/min

**Fig. 14:**
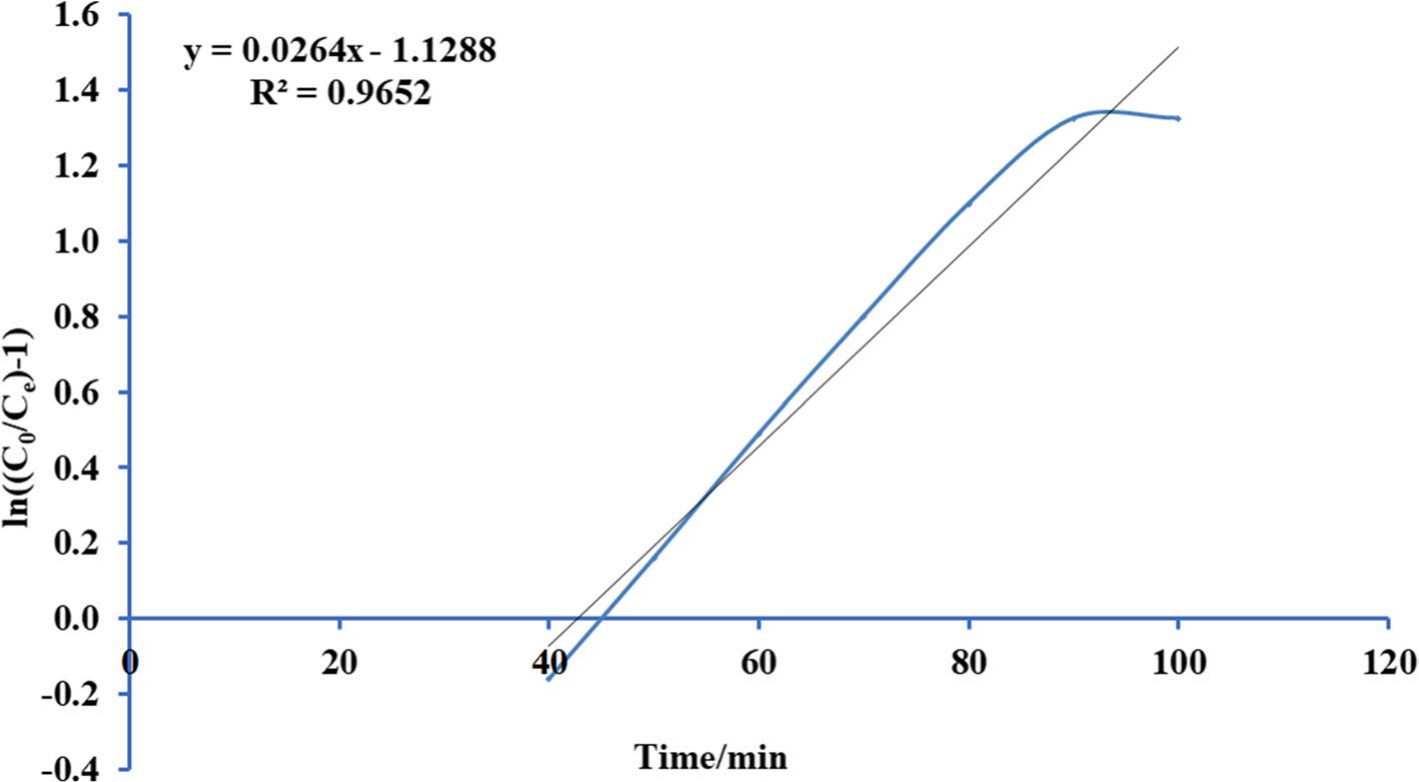
Plot Thomas mathematical model for ammonia adsorption by bed column at flow rate 15 ml/min

**Fig. 15:**
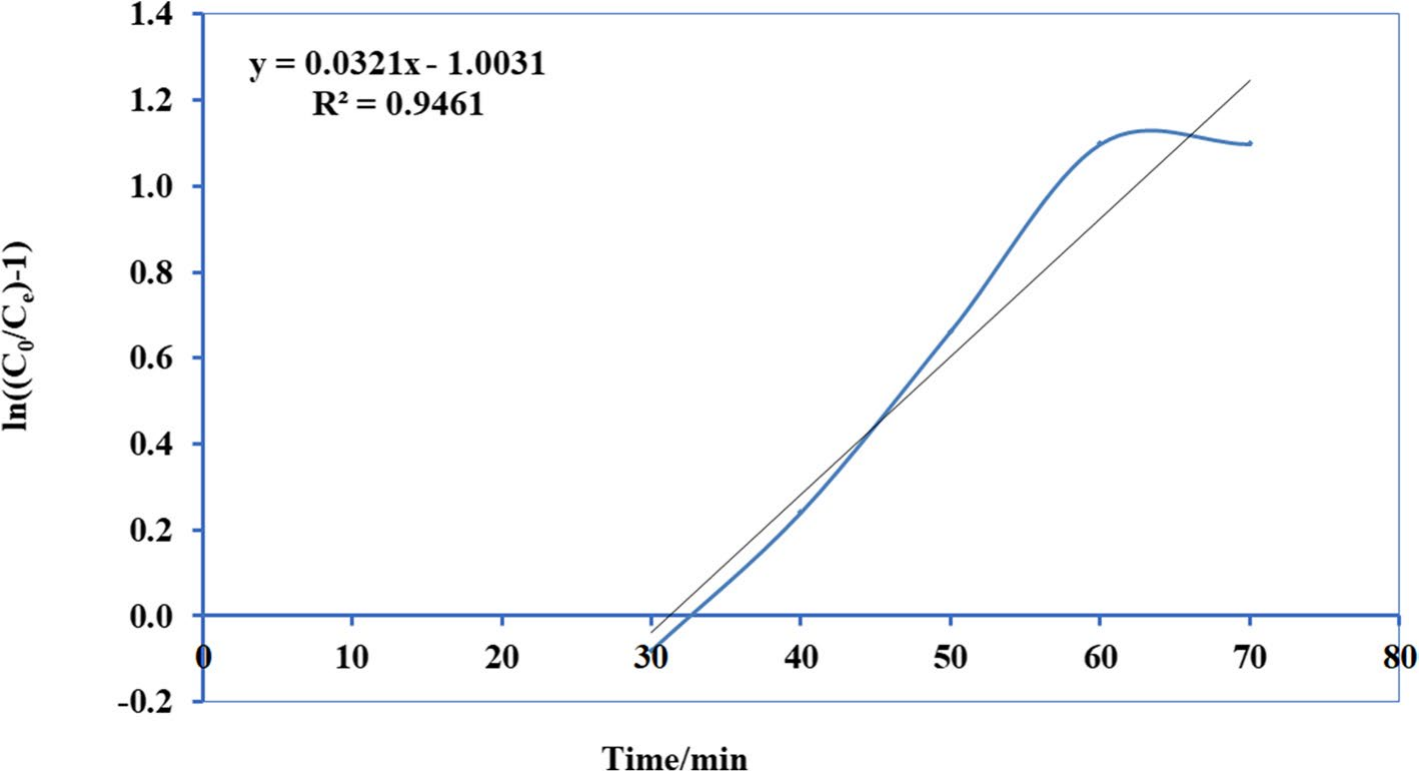
Plot Thomas mathematical model for ammonia adsorption by bed column at flow rate 20 ml/min

**Fig. 16:**
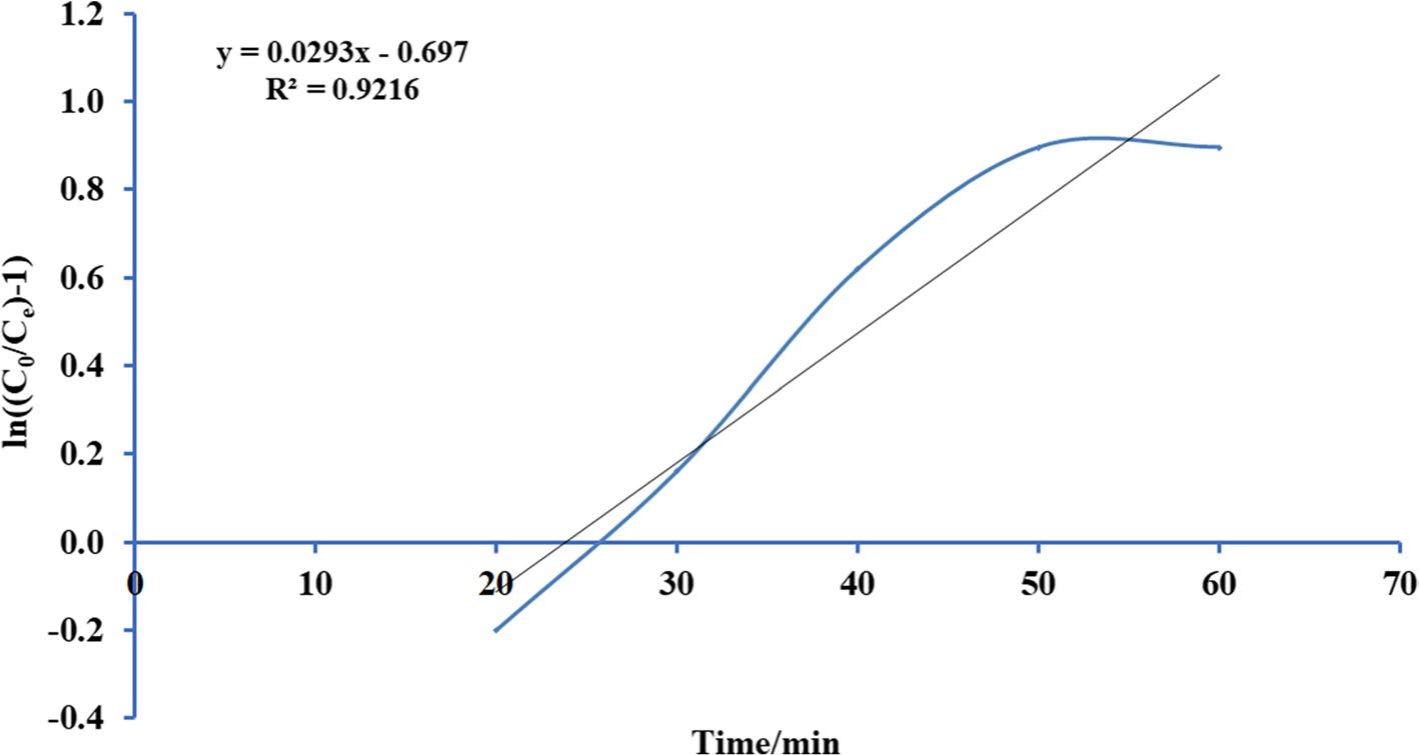
Plot Thomas mathematical model for ammonia adsorption by bed column at flow rate 25 ml/min

Thomas’s paradigm is the simple and generally utilized paradigms reported by many researchers [52–54]. Thomas model was adapted from the kinetics of the first-order reaction of adsorption model which is expressed in linear form equation as:

12 $$Ln\left[\left({C}_{0}/{C}_{e}\right)-1\right]=\left(\frac{{ M {Q}_{\mathrm{max}} K}_{Th}}{F} \right)-\left({K}_{Th}{C}_{0} \right)t$$

where *K*_Th_ is the Thomas model constant (l/mg.h), *Q*_max_ is the maximum uptake of solute (mg/g), *t* is the time (minutes), *M* is the mass of biosorbent, and *F* is the flow rate ml/min. *C*_0_ is the initial concentration of ammonia, and *C*_*e*_ is the concentration of ammonia in effluent solution. A conspiracy of Ln [(*C*_0_/*C*)-1] against *t* for a given flow rate of 10, 15, 20, and 25 ml/min can be applied to calculate the model constants. Figures 13, 14, 15, and 16 show the linear nature of the model yielding a virtuous fitting for the investigational results at all flow rates with high correlation coefficients (*R*^2^). The limitations of the Thomas model evaluated at the four rates of flow are reported in Table 6 which showed that adsorption capacity diminished with growing flow rate.

**Table 6:** Yoon and Nelson model parameters

Flow rate	*K*_YN_	*R*^2^	*t*_0.5_ mg/l
10	0.0356	0.987	64.2
15	0.0264	0.965	42.8
20	0.0321	0.946	31.2
25	0.0371	0.902	29.8

#### Yoon and Nelson’s Model

**Fig. 17:**
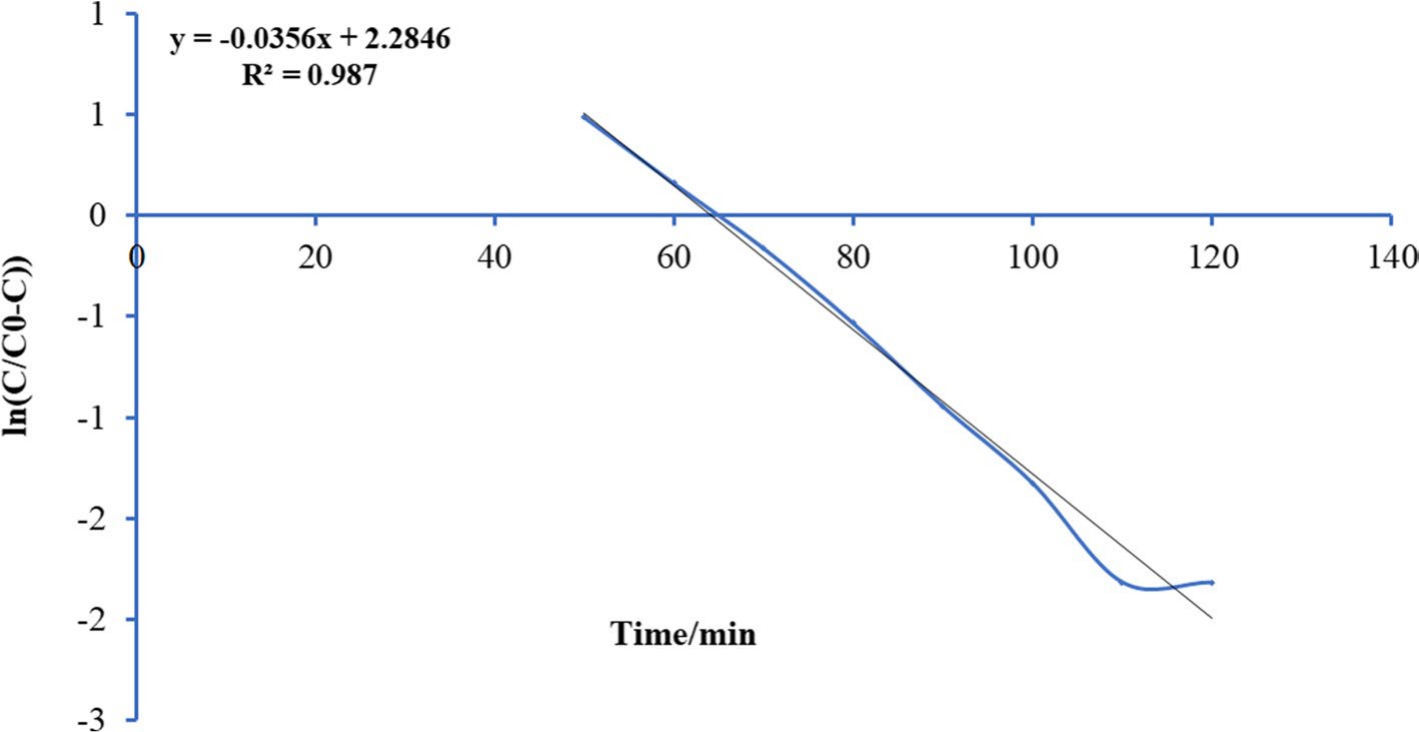
Plot Yoon and Nelson mathematical model for ammonia adsorption by bed column at flow rate 10 ml/min

**Fig. 18:**
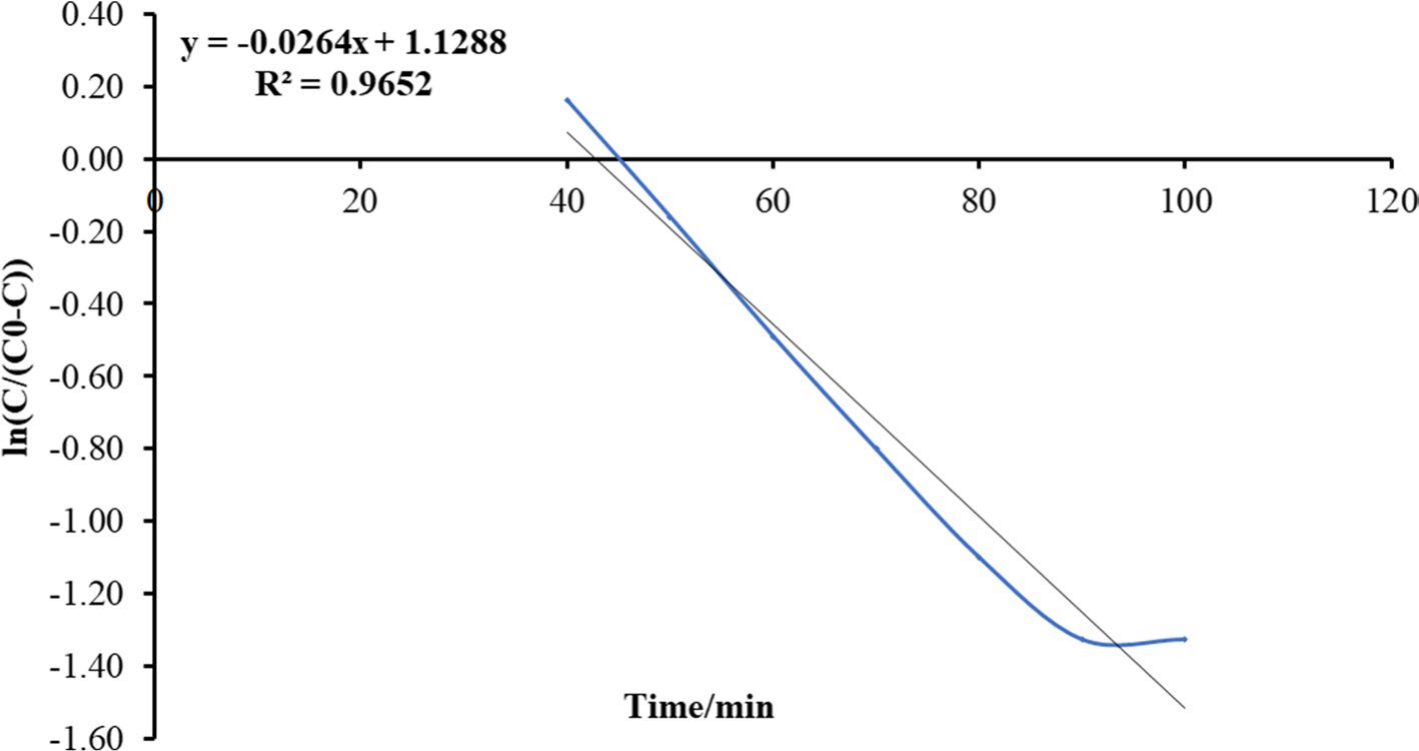
Plot Yoon and Nelson mathematical model for ammonia adsorption by bed column at flow rate 15 ml/min

**Fig. 19:**
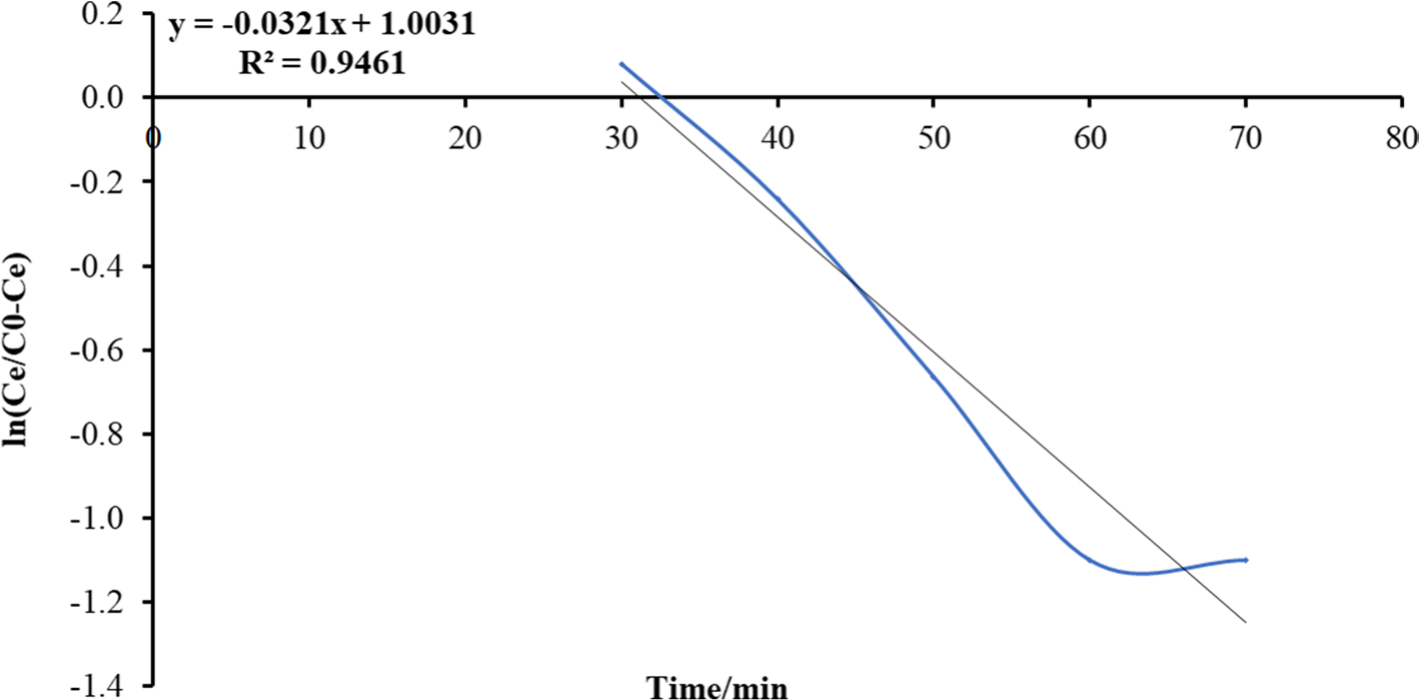
Plot Yoon and Nelson mathematical model for ammonia adsorption by bed column at flow rate 20 ml/min

**Fig. 20:**
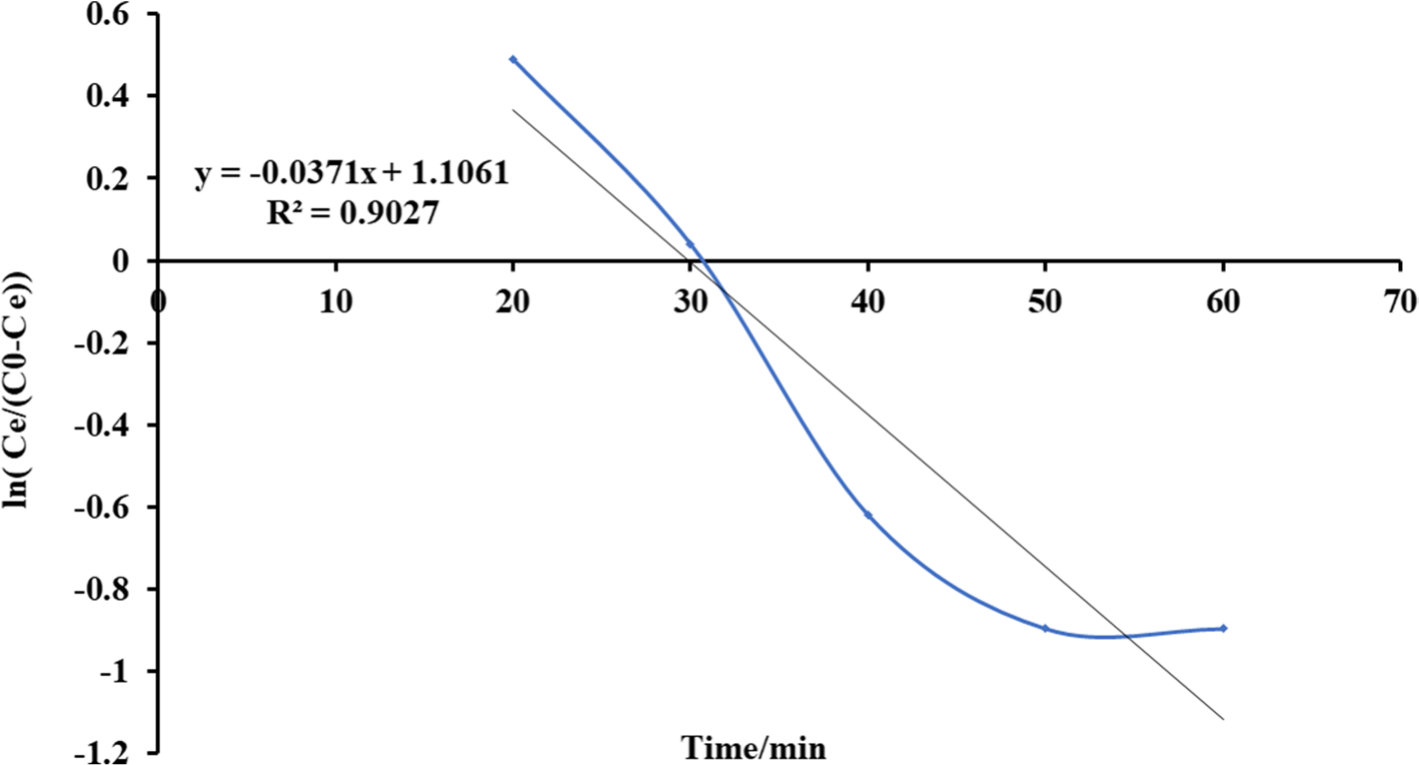
Plot Yoon and Nelson mathematical model for ammonia adsorption by bed column at flow rate 25 ml/min

This pattern estimates the possible decrease of the rate of adsorption which is directly proportional to its adsorption action; this model can be articulated as the following equation:

13 $$\mathrm{ln}\left(\frac{Ce}{{C}_{0}-{C}_{e}}\right.={K}_{YN}*t-\left({t}_{0.5}*{K}_{YN}\right)$$

where *C*_0_ (mg/l) is initial load, *C*_*e*_ (mg/l) is the load at time *t*, *K*_YN_ (1/min) is the rate constant of velocity, and *t*_0.5_ (min) is the revolution band for 50% of ammonia being adsorbed by adsorbent (Figs. 17, 18, 19, 20).

#### Bohart-Adams Model

**Fig. 21:**
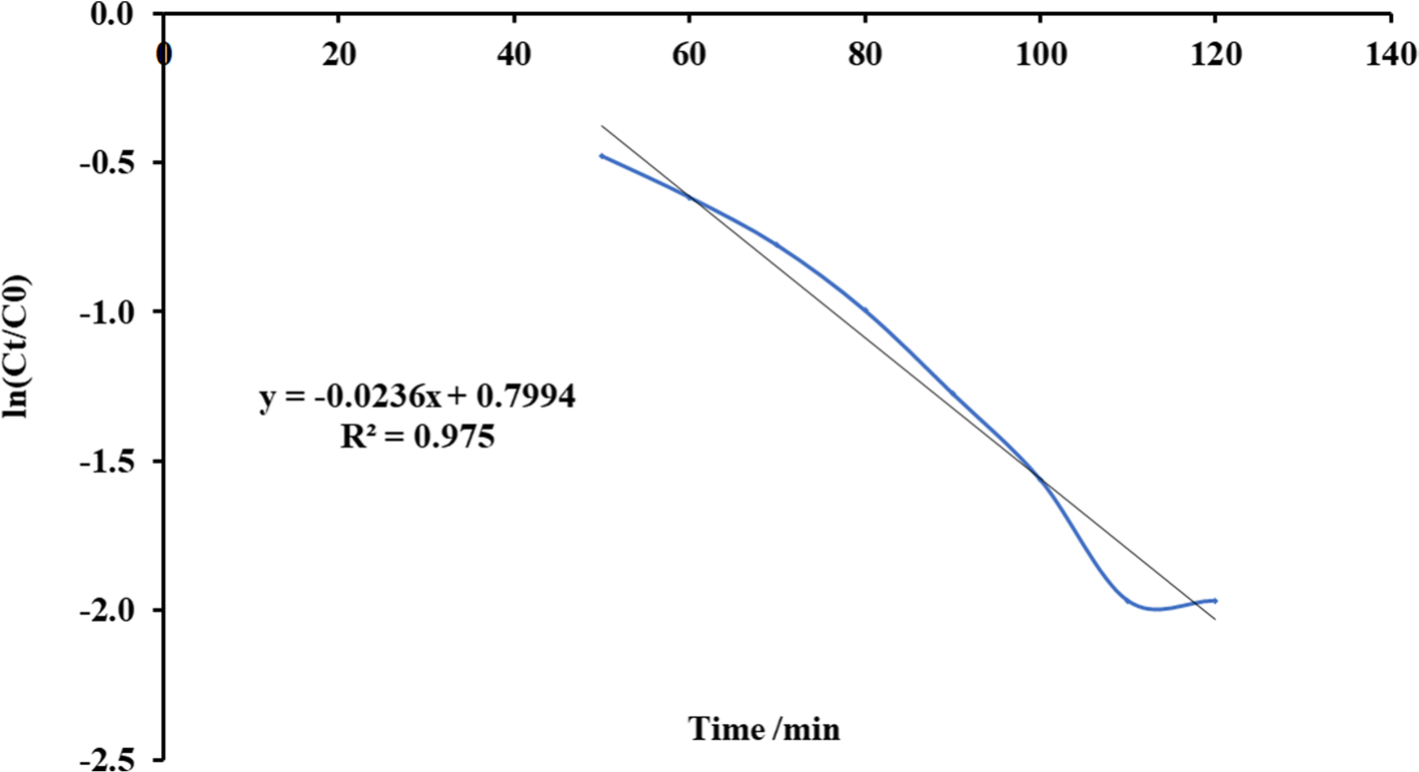
Plot Bohart-Adams mathematical model for ammonia adsorption by bed column at flow rate 10 ml/min

**Fig. 22:**
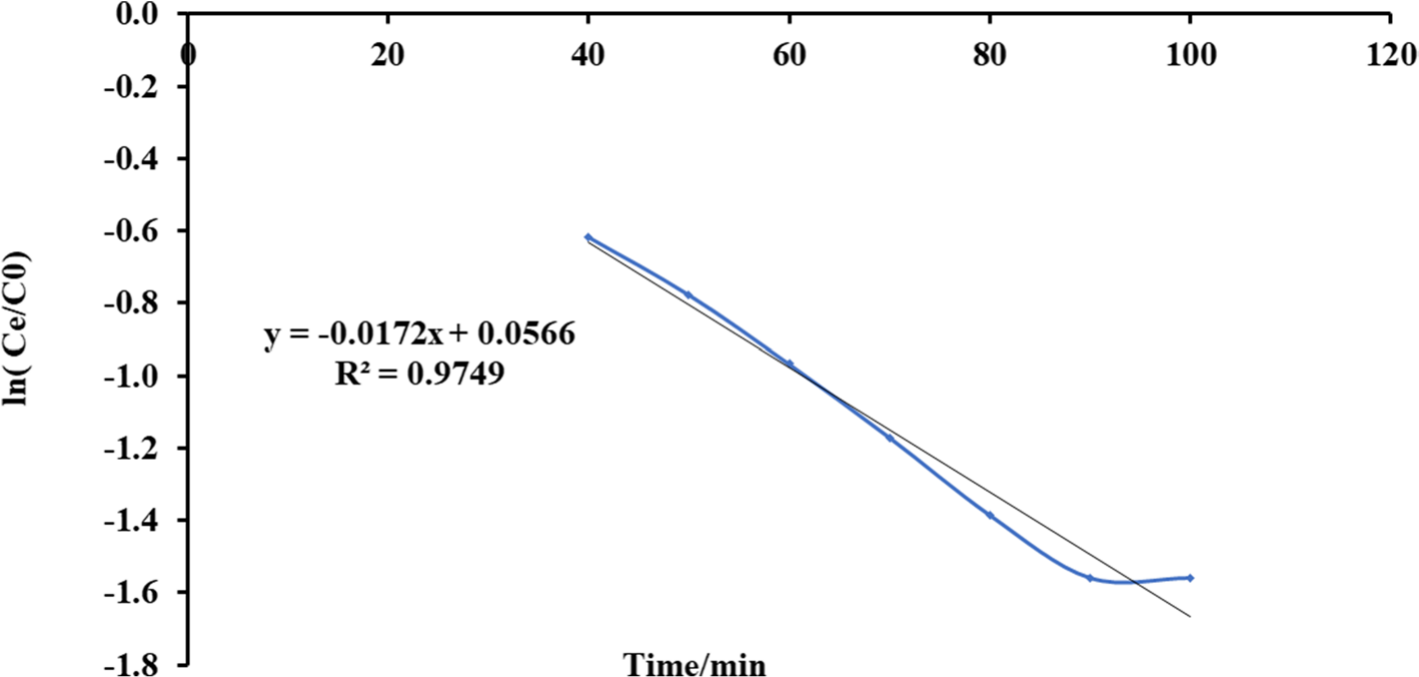
Plot Bohart-Adams mathematical model for ammonia adsorption by bed column at flow rate 15 ml/min

**Fig. 23:**
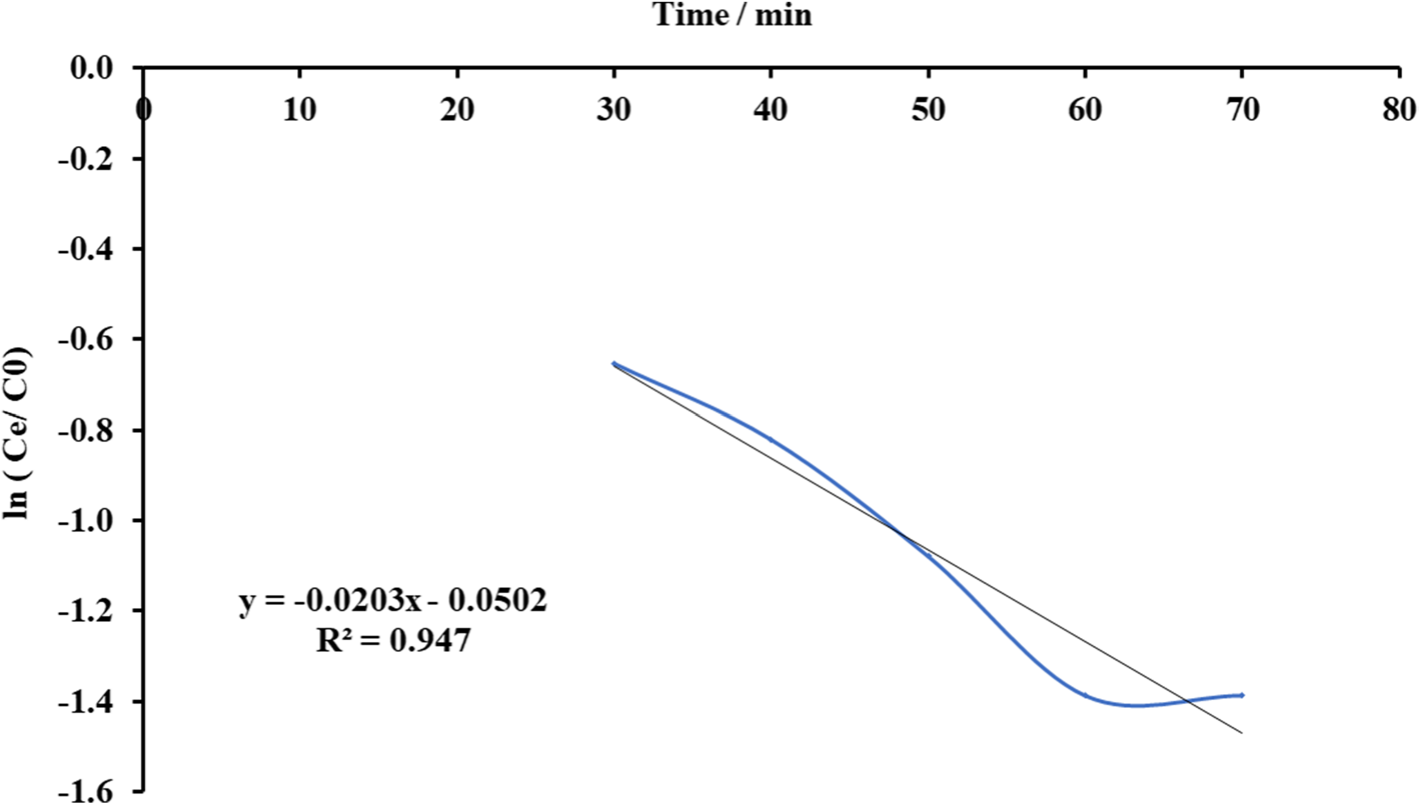
Plot Bohart-Adams mathematical model for ammonia adsorption by bed column at flow rate 20 ml/min

**Fig. 24:**
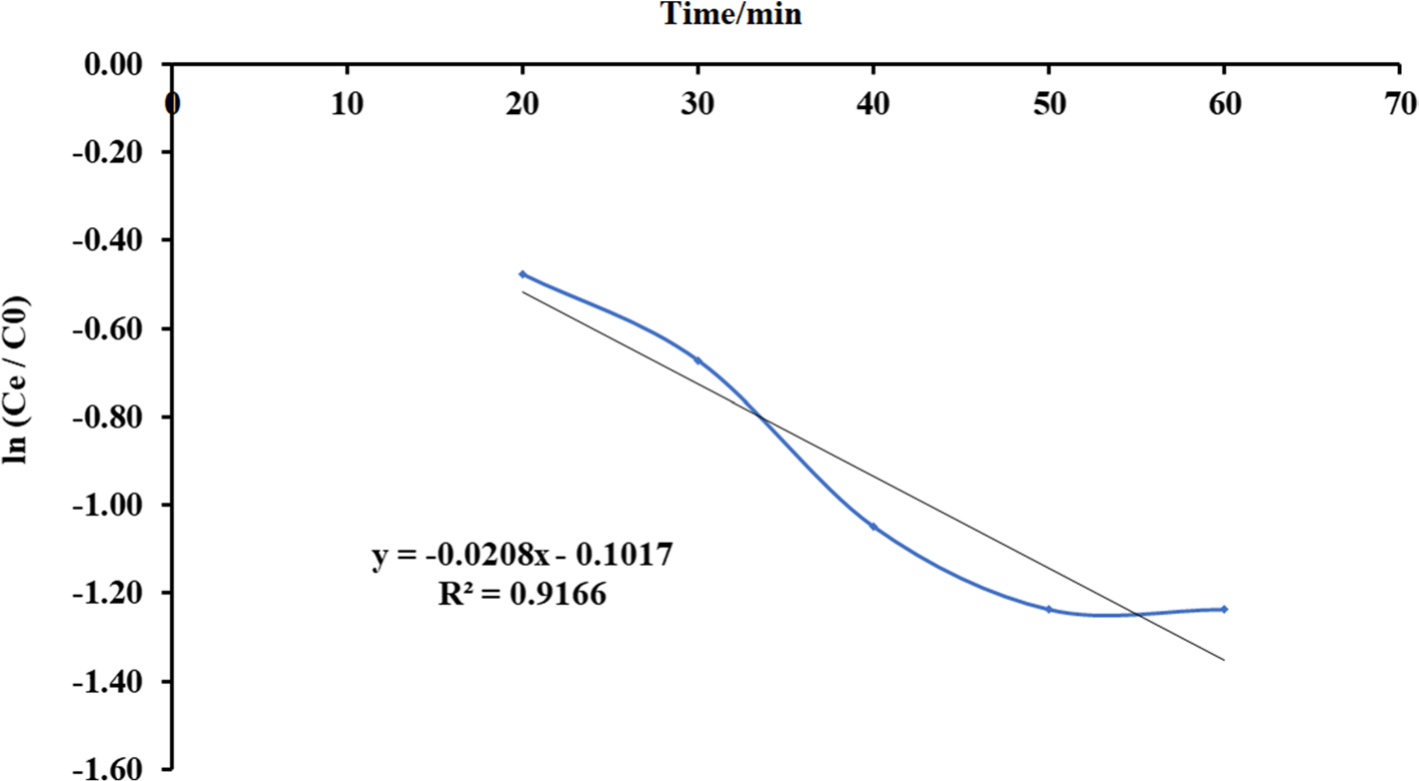
Plot Bohart-Adams mathematical model for ammonia adsorption by bed column at flow rate 25 ml/min

The Bohart-Adams model shows that the adsorption rate is directly proportional to the adsorbent power and concentration used. The equation to the model is defined below:

14 $$\mathrm{ln}\left({~}^{{C}_{t}}\!\left/ \! {~}_{{C}_{e}}\right.\right)={K}_{AB}{C}_{e}t-{K}_{AB}{N}_{0} {~}^{Z}\!\left/ \! {~}_{F}\right.$$

where *C*_0_ (mg/l) is primary load, *C*_*e*_ (mg/l) is the load at time *t*, *K*_AB_ (l/mg min) is constant of Bohart-Adams kinetic, *N*_o_ (mg/l) is capacity load, *Z* (cm) stands for divan penetration, and F (cm/min) is obtained by distributing the rectilinear speed of the rate flow with an expanse of the column (see Figs. 21, 22, 23, 24 and Table 7).

**Table 7:** The Bohart-Adams model parameters

Flow rate	*K*_AB_	*R*^2^	*N*_0_ mg/l
10	0.0024	0.975	8.5
15	0.0017	0.974	12.7
20	0.0020	0.947	12.8
25	0.0021	0.916	18.3

### Regeneration

The efficiency of biosorbent for ammonia removal decreases when it is applied for a long period, mainly because biosorbent gets saturated with NH_3_. Regeneration of NH_4_^+^ sorbent is a significant step in wastewater treatment for reuse of biosorbent and decreases treatment cost. Two categories of renewal were reported by the researcher: chemical and biological regeneration. In our research, chemical regeneration will be covered in detail. Chemical regeneration is supported by using acid (e.g., HCl, H_2_SO_4_) or alkali (e.g., NaOH with NaCl or CaCl_2_) chemicals. Chemical regeneration was reported in several studies [55–58]. The most used rejuvenation compounds are NaCl and HCl. In NaCl regeneration process, Na^+^ ion is exchanged with NH_4_^+^ ion which is loaded on biosorbents. Similarly, in HCl regeneration, H^+^ ions are exchanged with NH_4_^+^ ions which are loaded on biosorbents as exposed in the subsequent equivalences:

15 $$NaCl+Sorbent-{NH}_{4}^{+}\to {NH}_{4}Cl+Na-Sorbent$$

16 $$HCl+Sorbent-{NH}_{4}^{+}\to {NH}_{4}Cl+H-Sorbent$$

In this study, five loading and four rejuvenation sequences were carried out. *Eichhornia crassipes* powder (ECP) loaded with ammonia was regenerated with 60 g/l of NaCl medium at pH 12 with a flow rate of 10 ml/min. Ammonium ions (NH_4_^+^) are replaced by Na^+^ ions; then it converts to NH_3_ at high pH according to the next equations:

17 $$NaCl+ECP-{NH}_{4}^{+}\to {NH}_{4}Cl+Na-ECP$$

18 $${NH}_{4}Cl+{}^{-}OH\to {NH}_{3}+{}^{-}Cl+{H}_{2}O$$

**Fig. 25:**
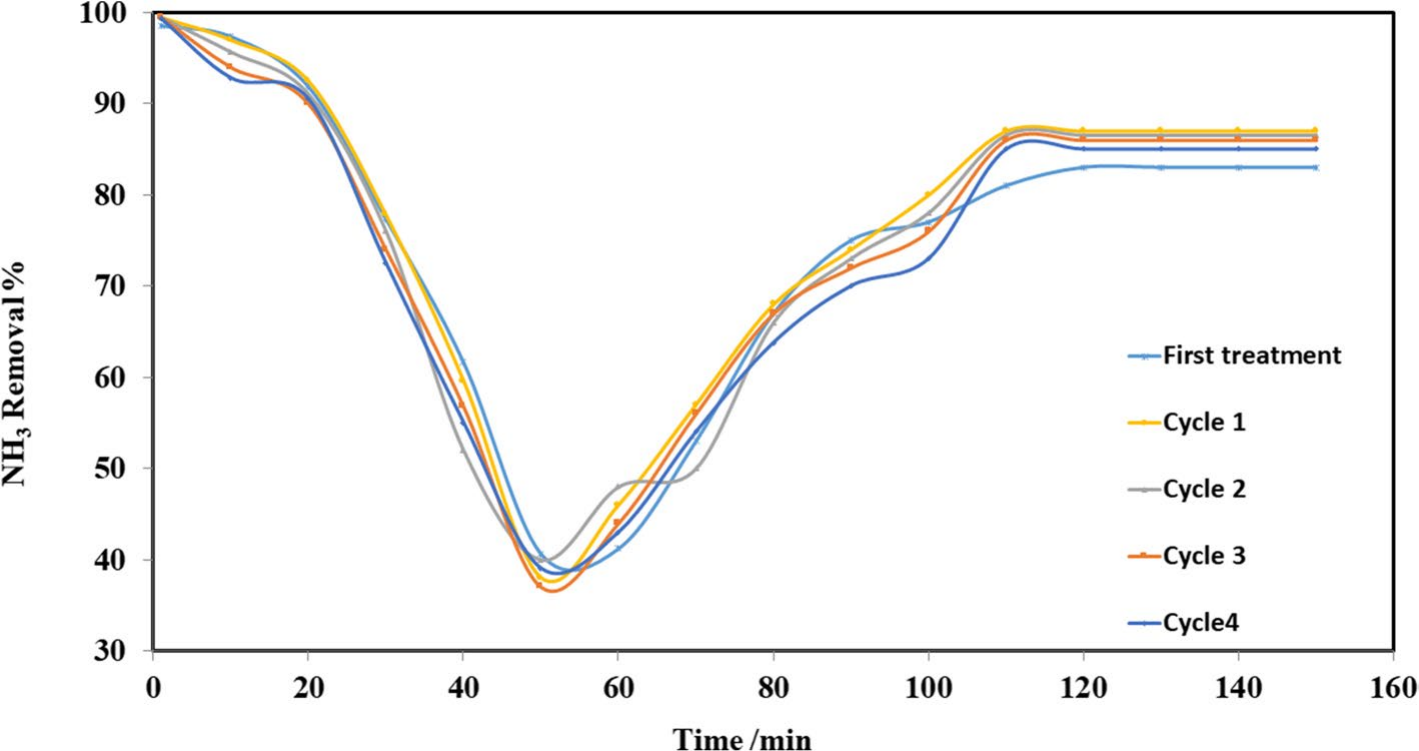
Adsorption recycles of ECP for ammonia removal

**Fig. 26:**
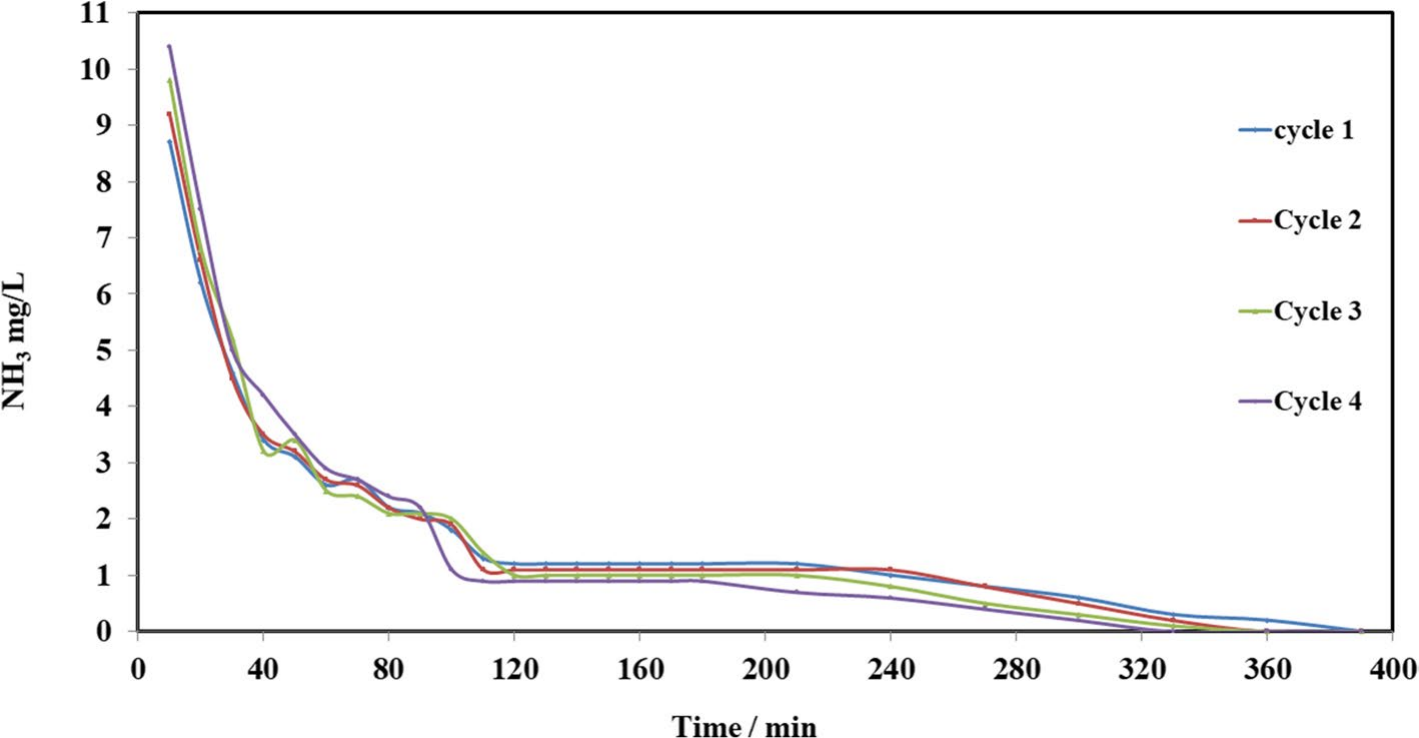
Ammonia elution by NaCl solution at pH 12 in a column system

ECP was washed with distilled water and dried at 80 °C to limit the loss in weight after five cycles; loss of weight was 15%. Elution efficiency (E) is considered from the following equation:

19 $$E\left(\%\right)=\left({M}_{d}/{M}_{biosorbent}\right)\times 100$$

where *M*_*d*_ (mg) is the mass of ammonia desorbed which was designed from the elimination result (*C* (mg/l) vs time/min). Figure 25 shows that NH_3_ removal % increased after the first regeneration due to Na^+^ ions which have activated ECP by converting it into ionic Na^+^ forms. When the regeneration cycle was repeated, NH_3_ removal % slightly decreased in a subsequent adsorption process. Figure 26 shows high regeneration (desorption) efficiency of ammonia with NaCl medium at pH 12.

### Case Study

The collected wastewater from the Sabal drain was subjected to a complete analysis rendering to the standard method as publicized in Table 8, the dealing with the collected wastewater utilizing the basin in addition to column methods was achieved, and the results are illustrated in Tables 8 and 9.

**Table 8:** Drainage wastewater characterization before and after treatment using ECP by basin adsorption method

Parameter	Unit	Raw wastewater	Treated wastewater	Removal %	Permissible limits
pH	-	7.55	7.36	-	7–8.5
COD	mg/l	358	89	75.2	> 30
BOD	mg/l	169	72.6	57	> 20
TDS	mg/l	880	17.8	97.9	> 500
Chloride (Cl^−^)	mg/l	260	10.75	95.8	-
Sulfate (SO_4_^2−^)	mg/l	146	25.00	82.8	> 200
Ammonia	mg/l	7.1	0.9	87	> 0.5
Nitrates (NO_3_^−^)	mg/l	20.8	1.1	93	> 45
Nitrite (NO_2_)	mg/l	0.4	0.02	95	> 0.4
Phosphate (PO_4_^3−^)	mg/l	1.6	1.4	12.5	> 1
Silica (SiO_2_)	mg/l	0.5	0.4	20	-
Iron (Fe^3+^)	mg/l	5.2	3.0	42	> 1
Manganese (Mn^2+^)	mg/l	0.23	0.09	60.8	> 0.5
Copper (Cu^2+^)	mg/l	0.26	0.20	23	> 0.2
Zinc (Zn^2+^)	mg/l	0.05	0.04	20	> 1.5
Fluoride	mg/l	0.02	0.02	0	> 1
Aluminum	mg/l	0.10	0.07	30	> 1.5
Lead	mg/l	0.10	0.05	50	0

**Table 9:** Drainage wastewater characterization before and after treatment using ECP by column adsorption method

Parameter	Unit	Raw wastewater	Treated wastewater	Removal %	Permissible limits
pH	-	7.58	7.52	-	7–8.5
COD	mg/l	377	74.2	80.3	> 30
BOD	mg/l	162	56.7	65	> 20
TDS	mg/l	863	12.7	98.5	> 500
Chloride (Cl^−^)	mg/l	447	15.3	96.5	-
Sulfate (SO_4_^2−^)	mg/l	152	25.00	83.5	> 200
Ammonia	mg/l	6.3	0.37	94.1	> 0.5
Nitrates (NO_3_^−^)	mg/l	24.5	1.2	95.1	> 45
Nitrite (NO_2_)	mg/l	0.6	0.02	96.6	> 0.4
Phosphate (PO_4_^3−^)	mg/l	0.6	0.2	66.6	> 1
Silica (SiO_2_)	mg/l	5.7	3	47.3	-
Iron (Fe^3+^)	mg/l	0.24	0.1	58.3	> 1
Manganese (Mn^2+^)	mg/l	0.22	0.1	54.5	> 0.5
Copper (Cu^2+^)	mg/l	0.1	0.07	30	> 0.2
Zinc (Zn^2+^)	mg/l	0.2	0.1	50	> 1.5
Fluoride	mg/l	0.01	0.01	0	> 1
Aluminum	mg/l	0.10	0.05	50	> 1.5
Lead	mg/l	0.04	0.01	75	0

#### Wastewater Treatment Plant

**Fig. 27:**
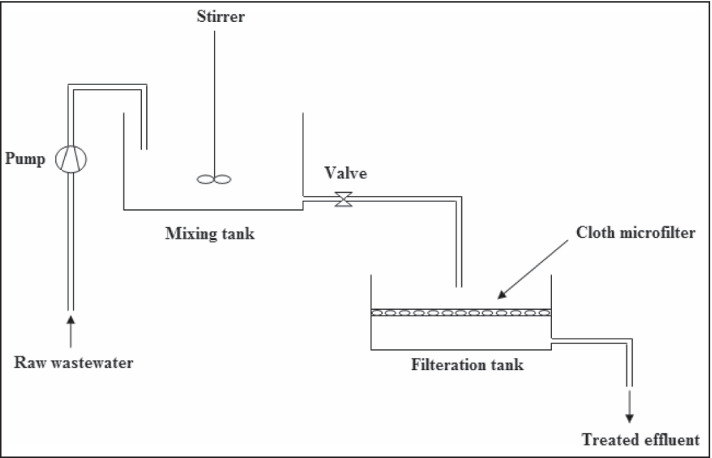
Wastewater treatment plant

Figure 27 shows a simple construction of the basin treatment plant for the adsorption method, its shelf life is 10 years, and it can treat 240 m^3^/day of polluted water; the plant consists of 2 basins with volume 30 m^3^ (its dimensions 4× 3× 2.5). Table 10 shows the affordable assessment for the building of the treatment factory. Table 11 shows the low-cost evaluation for the operating cost of the treatment plant. According to our previous study, the construction and consecutively expenses of the treatment can be premeditated proving that it is a low fee treatment; in comparison with the preceding studies, one study utilizing membrane technologies calculated a sum of 1.67 USD/m^3^ of the total cost; other researchers calculated the price of 1.974 $/m^3^; they utilize the electro-oxidation reactor, and our study presented a total cost ranging between 0.43 and 0.51 USD/m^3^ [42].

**Table 10:** Construction cost of treatment plant of basin method

Equipment	Total price (L.E.)
2 basins (30 m^3^) stainless steel	47,000
Pump (10 hp)	8000
Stirrer (5 hp)	9000
Connections and welding	2000
Total construction cost	66,000
Shelf life	10 years
Treated water	240 m^3^/day
Cost/m^3^	66,000/876000 = 0.08

**Table 11:** Running cost of treatment plant of basin method with ECP dose 5 kg/m^3^ of treated water

	Total price (L.E.)/m^3^
Energy	2
Materials	2
Worker	1
Cloth micro-filter	1
Maintenance	0.5
Cost/m^3^	6.50
Total cost/m^3^	6.58 LE

For the treatment of Sabal drain water by adsorption column, stainless steel column with depth of 5 m, diameter 2.8 m, total volume 30.77 m^3^, and side area 43.96 m^2^ will be used with a flow rate of 0.5 m^3^/min (30 m^3^/h), which can treat about 240 m^3^/day; this column needs about 4000 kg of ECP (16.6 kg ECP/m^3^) and density of ECP 133.3 g/l. Sabal drains discharge about 48000 m^3^/day to the Rossetti branch of the Nile River; to treat this quantity of water, we need 200 columns with volume of 30.77 m^3^. From Tables 10 and 11, treatment cost by basin adsorption method is about 6.5 L.E./m^3^, while treatment cost by column adsorption method (Tables 12 and 13) is about 10.5 L.E./m^3^, so the batch adsorption is the best method [59–62].

**Table 12:** Construction cost of only one adsorption column

Equipment	Total price (L.E.)
Cylindrical stainless steel column (30.77 m^3^)	22,000
Pump (10 hp)	8000
Connections and welding	4000
Total construction cost	34,000
Shelf life	10 years
Treated water	240 m^3^/day
Cost/m^3^	34,000/876000 = 0.04

**Table 13:** Running cost of adsorption column

	Total price (L.E.)/m^3^
Energy	2
Materials	6
Worker	0.5
Transportation	0.5
Cloth micro-filter	1
Maintenance	0.5
Cost/m^3^	10.50
Total cost/m^3^	10.54 L.E

## Conclusion

- Low-cost adsorbent, *Eichhornia crassipes* root powder (ECRP) was used for removing ammonia from synthetic and real drainage wastewater effluents.
- The batch method was employed for studying the behavior of some effective and restricted factors as pH; immersion period, dosage, and an original load of ammonia were premeditated at a temperature of 25 ± 2° C. Removal % of ammonia increase with growing the dosage of adsorbent, while the capacity of adsorption (*q*_*e*_) declines with growing up the dosage of the adsorbent. The optimal pH related to the extreme removal of ammonia was pH 6.
- Ammonia was stacked on the adsorbent rapidly through the initial 10 min, although equilibrium was achieved through 30 min. The maximum adsorption was 79% at optimum condition, initial concentration (10 mg/l), pH (6), interaction period (30 min), and ECRP dose (5 g/l).
- Langmuir constant (*R*_*L*_) magnitudes amongst 0 and 1 mean that the adsorptions are satisfactory, and a high value of K_L_ indicated strong bonding between ammonia and ECRP.
- Freundlich isotherm showed the removal of NH_3_ by ECRP yielded a line straight away. The standards of Freundlich constant (*n*) amongst 2 and 10 mean a decent outcome.
- The *R*^2^ in Langmuir isotherm was advanced than Freundlich isotherm for ammonia adsorption, so Langmuir isotherm is better fitted to the experimental data.
- Adsorption column was studied for removing ammonia from prepared wastewater; the flow rate consequence on the progression as; adsorption capacity declined with snowballing of flow rate.
- Thomas model yields a respectable fitting for the column investigational results at all flow rates with high *R*^2^ values; the parameters of the Thomas model showed that adsorption capacity decreased with increasing flow rate as 32.57, 31.82, 31.25, and 30.17 mg/g at a rate of flow of 10, 15, 20, and 25 ml/min, respectively.
- Yoon and Nelson’s model showed that *t*_0.5_ increased with the increase of flow rate, which was 29.8, 31.2, 42.8, and 64.2 min at a rate of flow 10, 15, 20, and 25 ml/min, respectively.
- Bohart-Adams model showed that saturation concentration increased with increasing the rate of flow which were 4.2, 6.2, 6.3, and 9.1 mg/l at a flow rate of 10, 15, 20, and 25 ml/min, respectively.
- The loaded powder of *Eichhornia crassipes* with ammonia was regenerated for five cycles with NaCl at pH 12. NH_3_ removal increased after the first regeneration due to the replacement of NH_4_^+^ by Na^+^, when the cycle was repeated, NH_3_ removal slightly decreased in a subsequent adsorption process, and NH_3_ removal at a steady state was 83, 87, 86.5, 86, and 85% for cycles 0, 1, 2, 3, and 4, respectively.
- For actual application, the adsorption technologies was applied to real drainage wastewater, Sabal drainage wastewater; removal of contaminants by batch adsorption method were COD (75.2%), BOD (57%), total dissolved solids (TDS) 97.9, chloride 95.4%, sulphate 82.8%, ammonia 87%, nitrates (NO_3_) 94.7, nitrite (NO_2_) 93%, phosphate 12.5%, silica 20%, iron 42%, manganese 60.8%, copper 23%, zinc 20%, free chlorine 7.1%, aluminum 30% and lead 50%; while pollutants removing by column method were COD (80.3%), BOD (65%) total dissolved solids (TDS) 98.5, chloride 96.5%, sulphate (83.5%), ammonia 94.1%, nitrates (NO_3_) 95.1, nitrite (NO_2_) 96.6%, phosphate 66.6%, silica 47.3%, iron 58.3%, manganese 54.5%, copper 30%, zinc 50%, free chlorine 25%, aluminum 50% and lead 60%.

## Data Availability

The datasets used and/or analyzed during the current study are available and exist in this manuscript.
